# Genome-wide analysis and transcriptomic profiling of the auxin biosynthesis, transport and signaling family genes in moso bamboo (*Phyllostachys heterocycla*)

**DOI:** 10.1186/s12864-017-4250-0

**Published:** 2017-11-13

**Authors:** Wenjia Wang, Lianfeng Gu, Shanwen Ye, Hangxiao Zhang, Changyang Cai, Mengqi Xiang, Yubang Gao, Qin Wang, Chentao Lin, Qiang Zhu

**Affiliations:** 1 Basic Forestry and Proteomics Center (BFPC), Fujian Provincial Key Laboratory of Haixia Applied Plant Systems Biology, Haixia Institute of Science and Technology, Fujian Agriculture and Forestry University, Fujian, 350002 China; 2 Department of Molecular, Cell & Developmental Biology, University of California, Los Angeles, California 90095 USA

**Keywords:** Moso bamboo, Auxin biosynthesis, Auxin transport, Auxin signaling, Gene expression

## Abstract

**Background:**

Auxin is essential for plant growth and development. Although substantial progress has been made in understanding auxin pathways in model plants such as Arabidopsis and rice, little is known in moso bamboo which is famous for its fast growth resulting from the rapid cell elongation and division.

**Results:**

Here we showed that exogenous auxin has strong effects on crown and primary roots. Genes involved in auxin action, including 13 *YUCCA (YUC)* genes involved in auxin synthesis, 14 *PIN-FORMED/PIN-like* (*PIN/PILS*) and 7 *AUXIN1/LIKE-AUX1* (*AUX1/LAX*) members involved in auxin transport, 10 auxin receptors (*AFB*) involved in auxin perception, 43 *auxin/indole-3-aceticacid* (*AUX/IAA*) genes, and 41 auxin response factors (*ARF*) involved in auxin signaling were identified through genome-wide analysis. Phylogenetic analysis of these genes from Arabidopsis, *Oryza sativa* and bamboo revealed that auxin biosynthesis, transport, and signaling pathways are conserved in these species. A comprehensive study of auxin-responsive genes using RNA sequencing technology was performed, and the results also supported that moso bamboo shared a conserved regulatory mechanism for the expression of auxin pathway genes; meanwhile it harbors its own specific properties.

**Conclusions:**

In summary, we generated an overview of the auxin pathway in bamboo, which provides information for uncovering the precise roles of auxin pathway in this important species in the future.

**Electronic supplementary material:**

The online version of this article (10.1186/s12864-017-4250-0) contains supplementary material, which is available to authorized users.

## Background

Auxin acts as a central organization hub in controlling plant growth and development [1, 2]. Auxin action can be achieved through different levels, mainly auxin concentration pathways including auxin biosynthesis and directional auxin transport, and auxin signaling pathways including auxin perception and signal transduction [3–5].

Although auxin biosynthesis is not fully understood in plants, genetic and biochemical studies have demonstrated that the endogenous plant auxin indole-3-acetic acid (IAA) is mainly synthesized by a two-step reaction: Trp is first converted to indole-3-pyruvate (IPA) by TRYPTOPHAN AMINOTRANSFERASE OF ARABIDOPSIS (TAA) and then IAA is produced by YUC flavin-containing monooxygenase family proteins [6]. YUC proteins that encode flavin monooxygenases catalyze a rate-limiting step of the IPA pathway [7]. In Arabidopsis, 11 YUC family proteins act redundantly and cooperatively at various growth and developmental stages [4, 6].

IAA mainly exists in the protonated form (IAA-H) in the apoplast, whereas the deprotonated form (IAA^-^) becomes dominant inside the plant cell due to the pH changes [8]. The IAA-H form of auxin freely enters the cell via diffusion, and the transport of its anionic form (IAA^-^) is mediated mainly by auxin influx transporters (AUX1/LAX proteins) and efflux transporters (ATP binding cassette B and PIN/PILS proteins) [5, 9]. In Arabidopsis, 4 amino acid permease-like family members (AtAUX1/LAX1–3) regulate auxin uptake from the apoplast [10], whereas 8 PIN proteins (AtPIN1-PIN8) and 7 PILS members (AtPILS1–7) are responsible for the polar pump-off of auxin and determine the direction of auxin flow through tissues [5]. The AtPIN-like family of proteins (AtPILS) was identified based on predicted topological similarities with AtPIN proteins [11]. As AtPIN proteins, AtPILS proteins contain the so-called InterPro auxin carrier domain, which is predicted to have auxin transport function in silico. In Arabidopsis, AtPILS proteins mainly localize in the endoplasmic reticulum (ER), and regulate intracellular auxin accumulation and the rate of auxin conjugation [11, 12]. The proper concentration of auxin throughout the plant body is achieved by auxin biosynthesis and polar auxin transport.

Synthesized auxin is distributed to the site of its action in a directional manner [5, 9]. The auxin regulatory module TIR1/AFB receptors-AUX/IAAs-ARFs, which is considered as the key components of the auxin signaling pathway in plant cells stimulates diverse auxin responses by coordinately controlling the expression of downstream genes [13]. In Arabidopsis, auxin binds to its receptor Transport Inhibitor Response 1 (TIR1), which belongs to a small gene family containing 5 additional members (AFB1–5). All of these 6 proteins redundantly function as nuclear auxin receptors [14–16]. TIR1/AFBs act as the specificity determiners for the SCF class of E3 ubiquitin ligases, which target substrate proteins for polyubiquitylation and subsequent degradation [17]. AUX/IAAs are negative regulators of the auxin signaling pathway [18]. Typically, AUX/IAA proteins have 4 conserved domains: domain I contains an ethylene responsive factor (ERF) associated amphiphilic repression motif and is required to recruit the transcriptional corepressor TOPLESS [19]; domain II is essential for auxin-induced AUX/IAA proteolysis [18]; and domains III and IV are involved in the homo- and hetero dimerization and interaction with the downstream auxin response factors (ARF) [20]. ARF proteins are a class of plant-specific B3-type transcription factors which mediate auxin-dependent transcriptional regulation [21]. In Arabidopsis, 23 ARF proteins act as either activators or repressors of the downstream auxin-responsive genes by binding to the auxin-responsive *cis-*element (AuxRE: [‘TGTCTC’]) [21]. The auxin receptor-AUX/IAA-ARF module precisely and sensitively controls the response of plant cells to auxin: without auxin, AUX/IAA proteins negatively regulate the abundance of ARFs, and subsequently the expression of auxin responsive genes; whereas upon elevated auxin level, the auxin-dependent ubiquitination and degradation of Aux/IAA proteins mediated by SCF^TIR1^ (TIR1 perceives auxin) releases ARF proteins to activate the transcription of auxin-responsive genes [13, 22].

Moso bamboo is one of the most important non-timber forest products in the world, due to its great economic, cultural, and environmental value [23]. Moso bamboo is famous for its fast-growing culms which were controlled by cell division and cell elongation [24]. Although the mechanisms that control plant cell number and size are not fully understood, phytohormones, especially auxin have important roles in the regulation and coordination of plant cell proliferation and elongation [25]. However, to our knowledge, no systematic study of the auxin pathway has been reported in moso bamboo. The genomic sequence of moso bamboo was recently released [26], providing an excellent opportunity to perform a comprehensive genome-wide analysis of the gene families related to auxin action. Here, a genome-wide search was carried out to identify auxin action-related genes in moso bamboo. A total of 13 *YUC* genes involved in auxin biosynthesis, 13 *PIN/PILS* and 7 *AUX1/LAX* family members involved in auxin transport, 10 putative auxin receptors involved in auxin perception, and 43 *AUX/IAA*s and 41 *ARF*s involved in auxin signaling were identified from the moso bamboo genome. Next we analyzed the phylogenetic relationships of auxin pathway orthologs from moso bamboo, Arabidopsis, and rice. Moreover, to generate a general overview of the auxin-response transcriptome in moso bamboo, we treated the plants with exogenous auxin and performed RNA-Sequencing (RNA-Seq) analyses. Our data provide a foundation for further investigations of the role of auxin in regulating moso bamboo development at the genetic and biotechnological levels.

## Methods

### Isolation of gene families related to auxin action in moso bamboo

The gene annotations and genomic sequences of moso bamboo were downloaded from the bamboo genome database (BambooGDB, http://www.bamboogdb.org/). To identify the members involved in auxin pathway in moso bamboo, we retrieved proteins of 11 AtYUCs, 8 AtPINs, 6 AtPILSs, 4 AtAUX1/LAXs, 6 AtTIR1/AFBs, 29 AtAUX/IAAs and 23 AtARFs from the Arabidopsis genome database, and used as query sequences against the moso bamboo database using basic local alignment search tool (BLAST) search. All sequences with an e-value ≤ 10^−11^ and a score value ≥100 were used as new queries for the 2nd cycle’s search to avoid missing additional orthologs. In addition, the derived sequences were used for further protein domain analysis using the Pfam program (http://pfam.xfam.org) and SMART (http://smart.embl-heidelberg.de/). To exclude the duplicated genes, all the candidates were aligned using the ClustalW program [27]. Information about the coding sequences, full-length sequences, and amino sequences were obtained from the bamboo genome database using the BLAST program.

### Gene structure, conserved motif, and protein information analyses

The genomic and cDNA sequences of each predicted gene were downloaded from the moso bamboo genome database, and their intron distribution patterns and intro-exon boundaries were analyzed using JBrowse software as previously described [28]. The conserved motif was derived using the NCBI conserved domain search (https://www.ncbi.nlm.nih.gov/Structure/cdd/wrpsb.cgi) or the online tool Pfam. The protein sequence was analyzed using DNAman software.

### Phylogenetic tree building and prediction of amino acid composition

Multiple sequence alignments of the auxin action-related proteins (YUC family, PIN/PILS family, AUX1/LAX family, AFB family, AUX/IAA family and ARF family) were performed using the ClustalW program with the default parameters [27]. The results were visualized using Editplus (https://www.editplus.com/). The phylogenetic tree was constructed from the protein sequences given above using MEGA6 program (http://www.megasoftware.net/mega.php) with the neighbor-joining (NJ) method, and the boot strap analysis was performed using 1000 replicates as described previously [29].

### Plant materials and auxin treatment

To determine the effects of auxin on the growth of moso bamboo, we germinated and grew bamboo seeds in the soil in a greenhouse at 26 °C with the photoperiod of 16 h light/8 h dark for 1 month. Various concentrations of naphthaleneacetic acid (NAA, 100 nM, 500 nM, and 5 μM) were sprayed the above-ground parts and watered the roots of the seedlings for 2 weeks at 2-day intervals, and pictures were taken for further statistical analysis using Image J software. At least 3 independent biological repeats were performed.

For materials used in RNA-Seq analysis, seeds were sterilized with chlorine gas for 3–5 h and were then put on Murashige and skoog (MS) agar to germinate and grow vertically. 1-month-old seedlings were sprayed with 5 μM NAA, 5 μM IAA or 5 μM Indole-3-butytric acid (IBA) for 4 h at 1-h intervals, and the root parts were dissected for RNA extraction. For the time course experiments, 1-month-old seedlings were sprayed with 5 μM NAA, and the roots were harvested at different time points (0, 2, 4, 6, 8 and 12 h) for further qPCR analysis.

### RNA extraction and RNA-Seq analysis

Total RNA from moso bamboo roots were extracted using the RNeasy Mini Kit (QIAGEN, China) according to the manufacturer’s instructions. 10 μg total RNA were used for RNA-Seq analysis using the Illumina Hiseq2500 Sequencer platform (BerryGenomics, China) with paired-end sequencing, and 3 independent biological repeats were performed. In total, 6 strand-specific RNA-Seq libraries were sequenced in this study using the deoxyuridine triphosphate (dUTP) method [30]. The paired-end reads were aligned to the moso bamboo genome using tophat-2.0.11 with anchor length more than 8 nt for spliced alignments [31]. Only reads that could be uniquely aligned were retained for subsequent analysis. The expression levels of each gene were normalized as fragments per kilobase of transcript per million mapped reads (FPKM) [32]. The *p*-value and false discovery rate (FDR) were calculated using the edgeR package developed in Bioconductor [33]. A fold change in the expression >1.5 and an FDR <0.01 were considered to be the threshold for differentially expressed genes (DEGs). To verify the RNA-Seq results, qPCR was used as previously described [34]. The primers used for qPCR are listed in the Additional file 1: Table S7. We used DAVID [35] to test the statistical enrichment of the differentially expressed genes in the KEGG pathways.

## Results

### Effects of exogenous auxin on moso bamboo

**Fig. 1:**
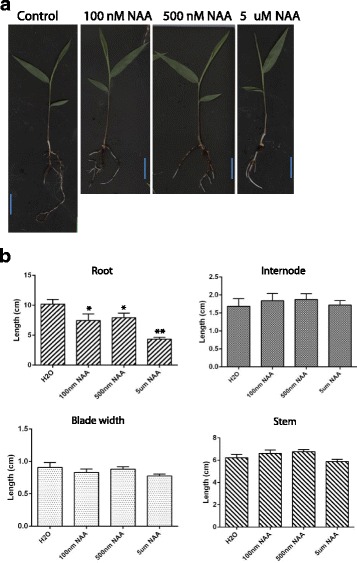
Exogenous auxin effects on moso bamboo seedlings. **a** 1-month-old moso bamboo seedlings grown in soil under greenhouse conditions were sprayed on the upper-part and watered on the root-part with various concentrations of auxin (100 nM, 500 nM and 5 μM). Pictures were taken after 2 weeks of treatment. **b** Statistical analysis of the seedlings from (**a**). The root length, internode length, whole stem length and blade width were statistically analyzed. *p < 0.05, **p < 0.001, *n* > 10

Auxin controls nearly every aspect of plant growth and development, but until now no reports showed its role in bamboo growth. To test its effects on the growth of bamboo seedlings, we treated the 1-month-old seedlings with various concentration of synthetic auxin NAA for another 2 weeks. Our results showed that bamboo root is sensitive to the exogenous NAA treatment, and NAA affects the root architecture in a dose-dependent manner, low concentration (100 nM and 500 nM) of NAA promoted the formation and growth of crown roots, while an inhibition of primary root and lateral root growth was observed at a higher concentration of auxin (5 μM) (Fig. 1a and b). Under our experimental conditions, we did not find significant differences in the above-ground plant architecture under our experimental conditions (Fig. 1a and b).

### Identification of gene families related to auxin action in moso bamboo

Extensive evidences showed that the auxin plays its function through controlling its homeostasis, transport and signaling (Additional file 2: Figure S1) [13, 36, 37]. As a first step to reveal the role of auxin in affecting bamboo root growth, we performed a genome-wide analysis to identify the auxin related genes in moso bamboo, including *YUC* family genes for auxin biosynthesis, *PIN/PILS* and *AUX1/LAX* families for polar auxin transport, *AFB* family for auxin perception, and *AUX/IAA* and *ARF* families for auxin signaling.

#### Auxin biosynthesis

Plant cells exhibit concentration-dependent auxin responses by tightly controlling cellular auxin levels [38]. Local auxin biosynthesis regulated by *YUC* family genes plays key roles in auxin accumulation [5, 9].

**Fig. 2:**
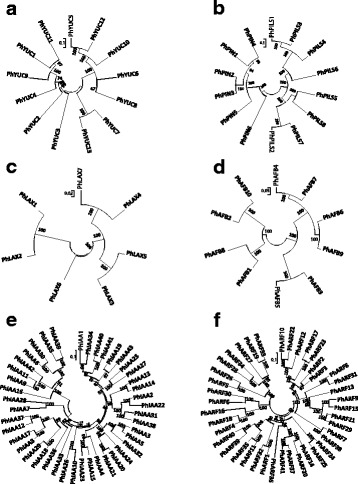
Genome-wide identification of auxin action-related gene families. Phylogenetic analysis of the identified PhYUC (**a**), PhPIN and PhPILS (**b**), PhLAX (**c**), PhAFBs (**d**), PhIAAs (**e**), and PhARFs (**f**). All the protein sequences were downloaded from bambooGBD, and the neighbour-joining method of the MEGA6 program was used to construct the phylogenetic tree. Bootstrap values from 1000 replicates are indicated at each branch

To identify *YUC* genes in moso bamboo, we searched the moso bamboo genome database using the Arabidopsis YUC proteins sequences as the queries. Combined with conserved domain analysis using Pfam or SMART, 13 *YUC* genes were identified in moso bamboo genome, and the phylogenetic analysis of predicted full-length YUC protein sequences were performed using neighbor-joining method (Fig. 2a). Our further search in the moso bambooGDB with these 13 *YUCs* did not identify additional *YUC* genes. We named these genes *PhYUC1*-*PhYUC13* based on the scaffold number (Fig. 2a, Additional file 1: Table S1). Analyses of the protein domains using the Pfam program showed that all of these proteins contained the conserved flavin-binding monooxygenase-like domain as reported in other species [39], whereas some PhYUC proteins have their own specific structures. For example, all PhYUC proteins except PhYUC3 contain FAD-binding motif [‘GxGxxG’]. PhYUC1, 4, 9 and 11 have the classical ATG-containing motif 1 [‘Y(x)7ATGEN(x)5P’], while PhYUC5, 6, 7, 8, 10, 12, and 13 share a conserved motif [‘D(x)4CI/NG(x)5P] in this region. The FMO identifying motif [‘FxGxxxHxxxY/F’], NADPH-binding motif [‘GxGxxG’], and ATG-containing motif 2 [‘(F/L) ATGY’] are conserved in all PhYUC proteins except PhYUC1 and PhYUC2 (Additional file 2: Figure S2A). These structural differences suggest the possible functional diversity among the PhYUC proteins.

The sizes of the deduced PhYUC proteins varied markedly. The open reading frame (ORF) lengths of the *PhYUC* genes range from 258 bp (*PhYUC11*) to 1656 bp (*PhYUC12*) with the predicted molecular masses varied from 9.4 to 170.6 kDa (Additional file 1: Table S1). To understand the gene structures of the *PhYUC* genes, the organization of exons and introns for each gene was obtained by comparing the cDNA sequences with the corresponding genomic sequences. The results showed that all members of the *PhYUC* family contained introns, ranging from 2 to 6 in numbers (Additional file 2: Figure S2B). The detailed information of the *PhYUCs* including the number of exons and introns, open reading frame lengths, translation lengths, molecular weights, conserved domains, and their putative subcellular localizations were listed in the Additional file 1: Table S1.

#### Auxin transport

After synthesis, auxin establishes the auxin gradient at the site for its function through auxin transport, a process which is controlled mainly by auxin efflux transporter PIN/PILS and influx transporter AUX1/LAX [5, 9]. To identify the *PIN* and *AUX1/LAX* members in moso bamboo, we used AtPIN/PILS and AtAUX1/LAX proteins from Arabidopsis as BLASTP queries to search for the moso bamboo orthologs in the bambooGDB. The Hidden Markov model (HMM) profiles (Pfam 01490: transmembrane amino acid transporter protein; Pfam 03547: membrane transport protein) were also employed to identify the PhLAX, PhPIN, and PhPILS protein families. A total of 6 *PhPIN* genes, 8 *PhPILS* and 7 *PhLAX* genes were identified and we further performed the phylogenetic analysis of their predicted full-length protein sequences using neighbor-joining method (Fig. 2b and c). All the *PIN* genes have similar intron/exon distribution patterns, and most of them have a large first exon that encodes the N-terminal transmembrane segments [5]. *PhPIN* genes contain 5–7 exons (Additional file 2: Figure S2C), and similar conserved exon/intron organization patterns have also been found in other plant species [40, 41]. As the case in Arabidopsis, PIN transporters in moso bamboo were classified into long PINs and short PINs based on the length of predicted proteins. The typical long PINs contained 547–611 aa (PhPIN1-PhPIN4), while the short PINs contain only 332 aa or 470 aa (PhPIN6 and PhPIN5, respectively) (Additional file 1: Table S2). Multiple sequence alignment results showed that the sequences of the N- and C-terminal transmembrane regions were highly conserved (Additional file 2: Figure S2D).

The ORFs of *PhPILS* genes range from 1023 bp (*PhPILS7*) to 1296 bp (*PhPILS1*) (Additional file 1: Table S2). The gene structures of the *PhPILS* genes showed that their exon/intron numbers were different: *PhPILS1*, *PhPILS3*, and *PhPILS4* contained 10 exons, *PhPILS2* and *PhPILS7* contained 9 exons, and *PhPILS5* and *PhPILS*8 contained 8 exons (Additional file 2: Figure S2E). Interestingly, *PhPILS6* contained only 1 exon (Additional file 2: Figure S2E), similarly to the *ZmPILS5* gene in maize [40]. As PhPIN proteins, multiple sequence alignment confirmed the high similarity among the PhPILS proteins. The results also revealed that the sequences of the N- and C- terminal transmembrane regions were highly conserved while the central hydrophilic region was variable (Additional file 2: Figure S2F), indicating the functional similarity among the members of the *PhPILS* family.

AUX/LAX proteins are required to establish the auxin gradient by mediating auxin influx transport [42]. In total, 7 auxin influx transporters (*PhLAX1*-*PhLAX7*) were identified in moso bamboo genome (Fig. 2c). The exon numbers of *PhLAX* ranged from 6 (*PhLAX6*) to 8 (*PhLAX4* and *PhLAX7*) (Additional file 2: Figure S2G), and the ORF lengths varied from 1431 bp (*PhLAX6*) to 1593 bp (*PhLAX3*) (Additional file 1: Table S3). Based on the multiple sequence alignment results, all members of this family shared conserved sequences (Additional file 2: Figure S2H). Moreover, all of these proteins were predicted to localize to the cell membrane (Additional file 1: Table S3), indicating that they have similar functions. The detailed information of these auxin transport-related genes is listed in Additional file 1: Tables S2 and S3.

#### Auxin signaling

In plants, the auxin receptor-AUX/IAA-ARF module consists of the key components involved in auxin signaling [3]. In moso bamboo, 10 putative auxin receptors that showed highly similar protein sequences were identified, and the phylogenetic tree of their predicted full-length protein sequences was generated using neighbor-joining method (Fig. 2d). The exon number of these genes ranged from 3 to 4 (Additional file 2: Figure S3A), the ORFs varied from 1632 bp (*PhAFB5*) to 1881 bp (*PhAFB6*) (Additional file 1: Table S4).

AUX/IAA proteins are the direct targets of auxin receptors [14, 43]. 43 AUX/IAA proteins were identified in moso bamboo genome, and the corresponding phylogenetic tree was generated using neighbor-joining method (Fig. 2e). The ORF lengths ranged from 252 bp (*PhIAA19*) to 2523 bp (*PhIAA30*) (Additional file 1: Table S5). The number of exons ranged from 2 to 18 (Additional file 2: Figure S3C). Sequence alignment analysis showed that most of the PhIAA proteins had typical domains I-IV. However, the diversity in the conserved domains was also observed (Additional file 2: Figure S3D). For example, the classical domain I [‘TELRLGLPG’] was found in PhIAA1, 34, 43, 40, 41, 25, and 27, while a similar domain [‘LR/K/T/ELXLXXPG’] was found in PhIAA2, 4, 8, 12–15, 17–18, 20–24, 29, 31, and 36–38. Moreover, in this region, a conserved domain [‘MRFK/RMR/KFRFEG’] was found in PhIAA6, 9, 11, 30, 35, 39, and 42. Whether these three domains have similar functions to that of the typical domain I needs to be further investigated (Additional file 2: Figure S3D). The other PhIAAs either lacked the domain I (PhIAA19 and PhIAA3) or had a poorly conserved domain I (PhIAA3, 5, 7, 10, 26, 28, and 32). Domain II [‘VGWPP’] was found in most of the PhIAAs, however, some members did not contain domain II (PhIAA19 and PhIAA34) or had poorly conserved domain II sequences. Moreover, we found a novel conserved domain in this region [‘LFGIXL’] with unknown function (Additional file 2: Figure S3D). Some members lacked either domain III (PhIAA19 and PhIAA22) or domain IV (PhIAA1, 34, and 28) (Additional file 2: Figure S3D).

Once the AUX/IAA protein was degraded, the ARF activities were released to activate downstream genes by binding to the AuxRE *cis*-elements [‘TGTCTG’] in their promoters. A total of 41 *ARF* members were identified in the bamboo genome, and the phylogenetic tree was built with the sequences of their proteins using neighbor-joining method (Fig. 2f). The ORF lengths of the *PhARFs* ranged from 1284 bp (PhARF3) to 3774 bp (PhARF10) (Additional file 1: Table S6). To understand the structural components of the *PhARF* genes, the exon and intron organizations of the genes were determined by comparing the cDNA sequences with the corresponding genomic DNA sequences. The coding sequences of all family members contain introns, and the number of exons ranged from 2 (*PhARF33*) to 17 (*PhARF1*) (Additional file 2: Figure S3E). To explore the structural diversity and predict the functions of ARFs in moso bamboo, a motif analysis was performed using the NCBI conserved domain search engine (https://www.ncbi.nlm.nih.gov/Structure/cdd/wrpsb.cgi). The results showed that all 41 putative PhARFs contained the highly conserved B3 DNA binding domain (DBD) (Additional file 2: Figure S3F and G). In Arabidopsis, the AtARF3, 13 and 17 proteins do not contain the carboxy-terminal dimerization domain (CTD domain) that is involved in the protein-protein interaction by dimerizing with AUX/IAA or with ARFs [21, 22]. Similarly to those proteins from Arabidopsis, the 12 ARF proteins in moso bamboo (PhARF2, 4, 6, 16, 18, 19, 25, 26, 28, 30, 33, 34, and 36) also lacked this conserved domain (Additional file 2: Figure S3F and G). In summary, the moso bamboo ARF proteins have the typical B3 DBD domains that were required for binding to the AuxRE *cis*- elements, while their structural variations implied that the moso bamboo genome changed significantly during its evolutionary history, indicating the functional diversities of these ARF proteins.

### Phylogenetic analysis of the gene families related to auxin action

To explore the possible roles of auxin action-related genes in moso bamboo and to understand their phylogenetic relationships, we constructed a phylogenetic tree using the proteins from rice, Arabidopsis, and moso bamboo. In general, the auxin action-related genes from bamboo showed high similarity to their orthologs from Arabidopsis and rice (Additional file 2: Figure S4).

The 38 members of YUC families in Arabidopsis, rice, and bamboo could be grouped into 3 classes. Of these proteins, PhYUC1, PhYUC9 and PhYUC11 belonged to class I (Additional file 2: Figure S4A) and were homologous to the genes *OsYUC5*, *OsYUC7*, and *OsYUC1* respectively. PhYUC2 and PhYUC4 belonged to class II, and the *PhYUC4* gene has two orthologs in rice *OsYUC9* and *OsYUC10*. In rice, *OsYUC9*, *OsYUC10*, and *OsYUC11* are highly expressed in the developing grains, and these genes are important for increases in the levels of IAA during grain development [44]. The highly close phylogenetic relationships of these genes indicated their potential function in bamboo grain development. Notably, the PhYUCs belonging to class III were not closely related to any rice or Arabidopsis *YUC* genes (Additional file 2: Figure S4A). This result probably reflects a diverging trend during the evolution of the YUC family members across different plant species.

Of the auxin receptor-like genes, the numbers of auxin receptor family in moso bamboo (10 members) was slightly expanded compared with that in Arabidopsis (6 members) or rice (8 members), and most of these genes are high closely related to the AFBs from these other two species (Additional file 2: Figure S4B). All the AFBs members can be grouped into 3 classes, and all AFBs from Arabidopsis and bamboo are grouped into class I and class II (Additional file 2: Figure S4B). Based on the phylogenetic tree analysis, *PhAFB10* and *PhAFB2* are closed to the Arabidopsis *TIR1*, which is well-characterized as the first-identified auxin receptor. It will be very interesting to determine the functions of these two genes in moso bamboo.

Although the PIN and PILS proteins are closely related, they belong to two separate groups in moso bamboo as in Arabidopsis and other species [11]. All *PIN* and *PILS* genes could be further divided into 3 subclasses (I-III) (Additional file 2: Figure S4C). In the PILS family, three pairs of PILS orthologs were identified between bamboo and rice: PhPILS8/OsPILS7a, PhPILS6/OsPILS2, and PhPILS4/OsPILS6 (Additional file 2: Figure S4C). In the PIN family, PhPIN1 belonged to the second group and was phylogenetically close to OsPIN10a and OsPIN10b, which are monocot-specific and have a long central hydrophilic domain. Based on the expression patterns of these genes in rice, they are involved in tillering [41, 45]. PhPIN2 and PhPIN3 are closely related to OsPIN1a, which is crucial for the negative phototropic curvature of the rice root [46]. PhPIN4 is the orthologs of the OsPIN1c and OsPIN1d from rice and *AtPIN1* from Arabidopsis, which have broad effects on plant development [45], indicating the important role of PhPIN4 in moso bamboo development. Interestingly, compared with the PIN subfamily, PhPIN6 is more closely related to the PILS subfamily (Additional file 2: Figure S4C).

The AUX1/LAX proteins could be divided into 2 major classes: PhLAX3, 4, 5, 6, and 7 belonged to class I, while PhLAX1 and 2 belonged to class II (Additional file 2: Figure S4D). *PhLAX3* and *PhLAX5* are closely related to *OsLAX2*, whereas *PhLAX4* and *PhLAX7* are closely related to *OsLAX4*; the *PhLAX1* and *PhLAX2* genes were closely related to *OsLAX1* (Additional file 2: Figure S4D). These results may reflect the occurrence of a whole-genome duplication event from moso bamboo and rice [26].

The AUX/IAA family is significantly expanded in moso bamboo (43 members) compared with those of Arabidopsis (29 members) and rice (24 members). As reported in other species [47, 48], AUX/IAA proteins in moso bamboo can be divided into 6 major classes (I-VI). Group I consisted of 12 PhIAA proteins, 6 OsIAA proteins, and 10 AtIAA proteins that form 12 sister pairs (4 PhIAA-OSIAA pairs, 4 PhIAA-PhIAA pairs, 4 AtIAA-AtIAA pairs). Group II contained 9 PhIAA proteins, 7 OsIAA proteins and 5 AtIAA proteins, which structure 7 sister pairs. Group III-VI contained 12 combined sister pairs. In total, 31 sister pairs were identified, and interestingly, no PhIAA-AtIAA pair was found (Additional file 2: Figure S4E). PhIAA15 is closely related to OsIAA1 in rice, and the overexpression of *OsIAA1* effectively inhibits root elongation and shoot growth [49]. PhIAA13 and PhIAA14 are phylogenetically close to OsIAA11, the overexpression of which leads to the loss of lateral roots in rice [50].

In regards to AUX/IAAs, the moso bamboo genome contains much more *PhARF* members (41 members) compared to the genomes of Arabidopsis (23 members) and rice (25 members) (Additional file 2: Figure S4F). The phylogenetic distribution results showed that the ARF genes from these three species could be divided into 5 major classes (I-V). Thirty-one members were clustered into class I (12 members from moso bamboo), 21 members were clustered into class II (9 members from moso bamboo), and 11 members (4 PhARF) and 15 members (7 PhARF) were clustered into classes III and IV respectively. Only two members from Arabidopsis (AtARF2) and rice (OsARF20) were classified into class V (Additional file 2: Figure S4F).

### Auxin treatment induces broad changes in transcriptional activity

**Fig. 3:**
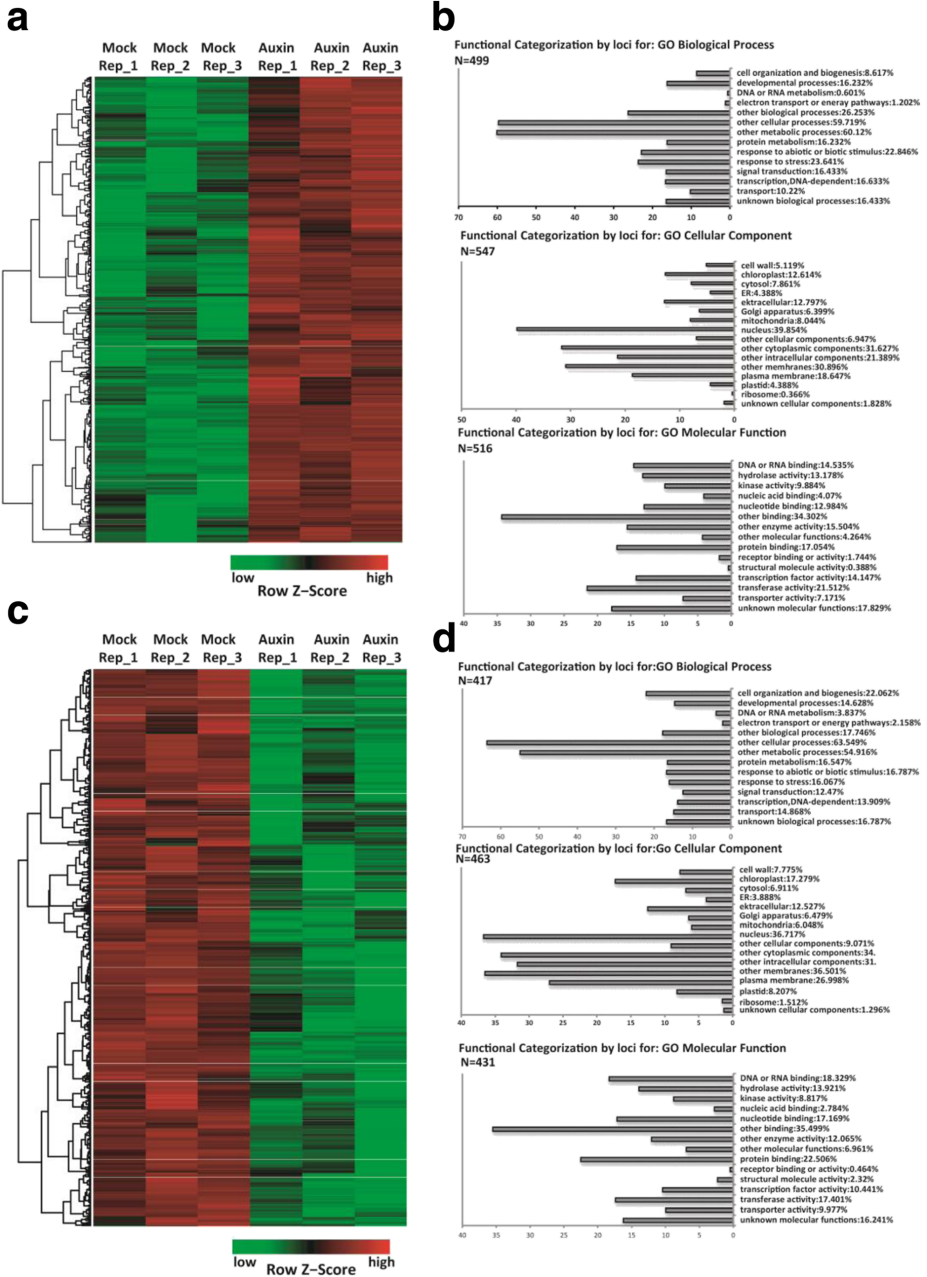
Overview of the auxin response genes in moso bamboo. Six samples from auxin treated and mock treated moso bamboo roots collected and were subjected to RNA-Seq analysis. Heatmap representation and hierarchical clustering of the differentially expressed transcript clusters for the up-regulated (**a**) or down-regulated (**c**) in response to auxin treatment are shown. Numbers on the nodes display major gene groups based on expression patterns. Color scale shows log2 signal intensity values. GO enrichment analyses for up-regulated genes (**b**) and down-regulated genes (**d**) in response to auxin. Best hits were aligned to the GO database, and most consensus sequences were grouped into three major functional categories: biological process, cellular component, and molecular function. Red indicates up-regulated genes; green indicates down-regulated genes

Under our experimental conditions, the roots of moso bamboo were sensitive to exogenous NAA treatment (Fig. 1). Our next step was to check the global gene expression changes in response to exogenous auxin by RNA-Seq. To optimize the conditions for this experiment, first we treated the bamboo seedlings with 5 μM NAA and the roots were harvested at different time points (0, 2, 4, 6, 8, and 12 h) for testing the expression patterns of selected auxin responsive genes. The genes we checked were:*Ph01003158G0110* and *Ph01000099G0730* for *Gretchen Hagen3* (*GH3* family); *Ph01000788G0760* for *the lateral organ boundaries domain* (*LBD family*); *Ph01004534G0130* for *small auxin-up RNA* (*SAUR* family); *Ph01000025G1600*, *Ph01000554G0550*, *Ph01000592G0620* and *Ph01000075G0200* for *AUX/IAA* family, whose orthologs in model plants such as Arabidopsis and rice were used as the markers for plant response to exogenous auxin treatment [51]. Our results showed that 5 μM NAA effectively changed the expression of these marker genes, and in most cases 4 h’ treatment had the strongest effects (Additional file 2: Figure S5). Therefore we treated bamboo seedlings with 5 μM NAA for 4 h and harvested the roots for RNA-Seq analysis, with the DMSO treated roots as the control. In general, with a cutoff of *P* < 0.05 and a fold change >1.5, we identified 991 down-regulated genes and 1288 up-regulated genes in the root 4 h after treatment with 5 μM NAA (Fig. 3, Additional file 3: Table S8). Our results from the Pearson correlation analysis showed that the correlations among all samples were quite high (>0.95), and the biological replicates could cluster well together (Additional file 2: Figure S6). To determine the reliability of the RNA-Seq results, we selected 28 differentially expressed genes related to auxin action and performed quantitative real time PCR (qRT-PCR). The results indicated a close correlation between RNA-Seq and qRT-PCR data (Additional file 2: Figure S7).

The upregulated- and downregulated-genes were classified into many functional categories, including stress response, developmental processes, cell organization and biogenesis, signal transduction, and other processes. Moreover, a significant number of the upregulated and downregulated genes were distributed across many cellular components, such as cell wall, membrane and chloroplast, which have a broad molecular function (Fig. 3). These results suggested that auxin stimulates broad transcriptional changes in the root of moso bamboo as in other species.

### Expression changes of genes related to auxin action in response to treatment with exogenous auxin

**Fig. 4:**
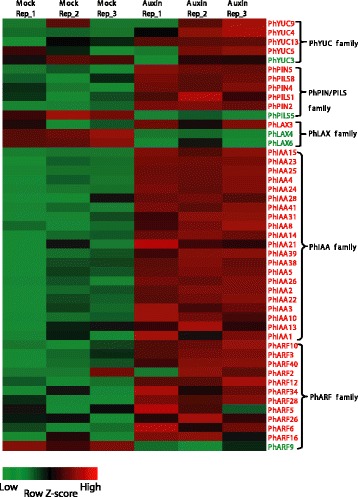
Heatmap representation of auxin action-related gene expression in response to auxin treatment. All auxin action-related genes including PhYUCs and PhPIN/PILS for auxin concentration and PhAFBs, PhIAAs, and PhARFs for auxin signaling were analyzed using the RNA-Seq data. The auxin action genes with significant changes at the transcriptional level are listed. The color scale shows log2 signal intensity values. Red indicates up-regulated genes; green indicates down-regulated genes

We further examined the auxin response of auxin biosynthesis, transport, and signaling pathway genes from RNA-Seq datasets, and the expressions of some randomly selected genes from different families were further confirmed by qRT-PCR (Additional file 2: Figure S8). Analysis of gene expression level (FPKM) showed that all these family genes have expressions in the root (Additional file 2: Figure S9A). Furthermore, results from our qRT-PCR analysis of selected DEGs from various families of auxin pathways in different tissues showed that these auxin-related genes were expressed in root, shoot and leaf, with different extent of expression in these tissues (Additional file 2: Figure S9B). In Arabidopsis, the *YUC1*, *2*, *4* and *6* genes were downregulated in response to the exogenous NAA treatment through the feedback pathway [52]. In moso bamboo, 5 of the 13 *PhYUC* members responded to elevated auxin levels: *PhYUC4*, *5*, *9* and *13* were significantly upregulated, and *PhYUC3* was downregulated (Fig. 4, Additional file 4: Table S9).

In Arabidopsis, the expression of most auxin transporter genes are positively regulated by auxin, contributing to faster auxin transport when endogenous auxin is elevated [53]. In bamboo, we found that 3 *PIN* genes (*PhPIN2*, *4*, *5*) and 3 *PILS* genes (*PhPILS1*, *5*, *8*) changed their expression in response to exogenous auxin treatment. Auxin stimulated the expression of *PhPIN2*, *4*, *5* and *PhPILS1*, *8*, while downregulated the expression of *PhPILS5* (Fig. 4, Additional file 5: Table S10).

In Arabidopsis roots, all *LAX* genes are upregulated by auxin treatment [51]. However, we found that in moso bamboo, the expression of 2 *PhLAX* genes (*PhLAX4* and *PhLAX6*) were downregulated by exogenous auxin treatment, whereas the transcriptional level of *PhLAX3* was upregulated (Fig. 4, Additional file 6: Table S11).

For auxin signaling pathway, auxin receptor genes did not changed at transcriptional level (Additional file 7: Table S12). The most pronounced effects of the plant response to auxin are the upregulation of *AUX/IAA* genes, and auxin signaling is activated by auxin-dependent degradation of proteins from this family [18]. In total, 21 *PhIAA* transcripts were upregulated by auxin in moso bamboo. Importantly, the transcription of *PhIAA23*, an ortholog of *AtIAA19* in Arabidopsis and acts as the key regulator in Arabidopsis lateral root formation [54], was strongly induced in response to exogenous auxin. This result is quite similar to the data from Arabidopsis and other plants, indicating that auxin-activated *AUX/IAA* gene upregulation is conserved in the plant kingdom (Fig. 4, Additional file 8: Table S13).

ARF proteins bind to the promoter elements and act as activators or repressors of downstream auxin responsive genes [21]. Of the 41 members of the ARF family identified in moso bamboo, 11 *PhARFs* were upregulated and 1 *PhARF* was downregulated by auxin (Fig. 4, Additional file 9: Table S14). Among the auxin responsive genes in Arabidopsis, *AtARF19,* an activator of auxin-dependent transcription is most sensitive to auxin at the transcriptional level. *AtARF7* and *AtARF19*, which are phylogenetically close, are considered to be the only ARF factors that are necessary and sufficient for auxin signaling in 7-d-old light-grown seedlings [55]. Supporting this point, *PhARF11,* the ortholog of *AtARF19,* and *PhARF13,* the ortholog of *AtARF7*, were strongly induced by auxin (Fig. 4, Additional file 9: Table S14). *AtARF2* acts as the communication node that links the ethylene and auxin signaling pathways [21], and its ortholog *PhARF42* in bamboo was strongly induced by auxin. All the auxin pathway genes that changed expression at transcription level were listed in the Additional file 10: Table S15.

It is likely that moso bamboo possesses similar conserved signaling pathways that stimulated the downstream genes as other species, such as Arabidopsis. On the other hand, different mechanisms in controlling the auxin concentration exist between moso bamboo and Arabidopsis, for example, the expression of some auxin transporter genes in bamboo went down, whereas all their orthologs in Arabidopsis were upregulated after auxin treatment. Future analyses of the expression patterns and subcellular localizations, as well as the functional characterization of these genes will unveil the specific mechanisms of controlling the auxin-concentration related pathways in moso bamboo.

### Identification of additional important auxin-responsive transcription factors

Except for the AUX/IAA family, 3 other gene families participate in the primary auxin response at the transcriptional level in Arabidopsis: *Gretchen Hagen3* (*GH3* family), *small auxin-up RNA* (*SAUR* family) and *the lateral organ boundaries domain* (*LBD* family) [51, 56, 57]. *GH3* genes encode a class of auxin-induced conjugating enzymes and are auxin responsive genes involved in regulating auxin homeostasis and response [58]. At least 7 *GH3* genes are strongly and rapidly induced by auxin in Arabidopsis [58]. Studies from Arabidopsis and the moss *Physcomitrella patens* suggested the evolutionarily conserved role for *GH3* proteins in regulating auxin homeostasis [59]. In the bamboo genome, we found 5 putative *GH3* genes with increased transcription in response to auxin (Additional file 11: Table S16). Among the early auxin response genes, the *SAUR* gene family has the largest number of member; it is estimated that around half of the Arabidopsis *SAUR* gene transcripts are rapidly upregulated by auxin, while a small number of this family members appear to be repressed by auxin [60, 61]. Recently, a genome-wide analysis of *SAUR* genes was performed in moso bamboo [62]. Of the 38 *SAUR* genes identified, 10 *SAUR* genes changed their expressions under our auxin treatment conditions, the expression levels of 7 of those genes increased, whereas 3 genes decreased (Additional file 12: Table S17). The *LATERAL ORGAN BOUNDARIES* (LOB) proteins, which contain a conserved LOB domain (LBD), are rapidly and specifically up-regulated by auxin treatment [55]. Our RNA-Seq results also showed that in moso bamboo, at least 9 putative *LBD* genes increased their transcripts in response to exogenous auxin treatment (Additional file 13: Table S18).

Based on the acid growth hypothesis, auxin stimulates protons pumping into the cell wall matrix, thereby helping to loosen the cell wall [63]. On the other hand, auxin may modulate cell wall properties by regulating the transcription of genes related to cell wall remodeling [64]. Consistent with this hypothesis, we found that a number of cell wall-related genes encoded structural cell wall proteins or enzymes changed their expressions after auxin treatment (Additional file 3: Table S8). In total, 51 genes, including expansins, xyloglucan endotransglycosylases (XTHs), and pectinmethylesterases (PMEs), were identified. Of these genes, 24 were upregulated and 27 were downregulated (Additional file 14: Table S19). Expansins were identified as the major cell wall-loosening agents [63]. In bamboo, at least 5 expansin-like genes were upregulated at the transcriptional level (Additional file 14: Table S19). Xyloglucan endotransglycosylase /hydrolases that cut and paste xyloglucans, and endoglucanases which hydrolyze glucosidic bonds, also contribute to cell wall loosening and cell expansion by modifying cell wall properties and by integrating new wall materials, respectively [63]. Supporting to this point, 3 glucanase-related genes changed in expression after auxin treatment (Additional file 14: Table S19). Some glycanases that were transcriptionally increased by auxin catalyze the hydrolyses of cell wall polysaccharides, and are involved in the auxin-induced changes in the mechanical properties of cell walls [65]. We found that 4 genes encoding glucanases changed in expression after auxin treatment (Additional file 14: Table S19). Class III peroxidases induce cell wall loosening and growth by elongation as well as cross-linking of cell wall components. It should be noted here that the expression levels of two putative peroxidase genes were also changed (Additional file 14: Table S19). These results suggest that auxin induces a broad range of transcriptional changes in cell wall property-related genes.

### Crosstalk of auxin and other phytohormones in moso bamboo

**Fig. 5:**
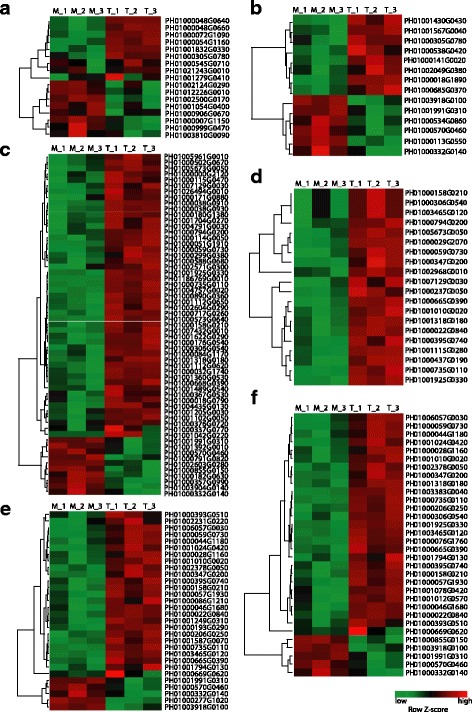
Cross-talk between auxin and other phytohormones. The hierarchical clustering of auxin-responsive genes that are associated with cytokinin (**a**), ethylene (**b**), Gibberellin (**c**), ABA (**d**), SA (**e**), and JA (**f**) were shown. M_1–3, mocked sample with 3 repeats; A_1–3, auxin treated samples with 3 repeats. Color scale shows log2 signal intensity values. Red indicates up-regulated genes; green indicates down-regulated genes

The crosstalk among hormone-regulated pathways was widely present in plant cells [37]. We further examined the interaction between auxin and other phytohormones. Cytokinin and auxin have long been recognized as crucial signaling molecules controlling plant growth and development [66]. Cytokinin is degraded by side chain cleavage through the action of the cytokinin oxidase/dehydrogenase (CKX) enzymes which are induced by auxin at the transcriptional level [67]. In moso bamboo, at least two putative cytokinin oxidases genes (*PH01000072G1090* and *PH01001279G0410*) increased their transcriptional expression in the presence of auxin (Fig. 5a, Additional file 15: Table S20). Cytokinin conjugation is an important process that maintains the subcellular levels of active cytokinin. For example, the interactions between glycosides and cytokinins which are catalyzed by cytokinin-*O*-glucosylation inactivate cytokinins. We found that the expression of *PH01021243G0010*, a putative ortholog of *cytokinin-O-glucosyltransferase 1* in maize, was upregulated. Type-A ARRs are negative regulators of cytokinin signaling in bamboo, one type-A ARR gene (*PH01000999G0470*) was upregulated by auxin (Fig. 5a, Additional file 15: Table S20). *PH01000007G1150* is an ortholog of *HAT22* which belongs to a family of cytokinin-regulated homeodomain zip (HD zip) class II transcription factors in Arabidopsis [68], and was activated by auxin at the transcriptional level in moso bamboo (Fig. 5a, Additional file 15: Table S20). Therefore auxin signaling pathways affect cytokinin synthesis, homeostasis, and other signaling pathways at the transcriptional level in moso bamboo as in other model plants like Arabidopsis and rice.

In total, 14 gibberellin acid (GA) related genes were responsive to auxin treatment in the moso bamboo root. Of these genes, 3 putative gibberellin-2-oxidases which are involved in GA degradation were downregulated. One putative Gibberellin-20-oxidase-2 which controls GA biosynthesis was upregulated (Fig. 5b, Additional file 16: Table S21). These results suggested that exogenous auxin controls GA levels through transcriptional regulation. A previous study in Arabidopsis showed that the effects of auxin on GA biosynthesis and signaling are complicated and depend on the concentration of exogenous auxin, duration of treatment or tissue type [51]. Therefore, more detailed analyses are needed to fully illustrate the crosstalk between auxin and GA.

The importance of auxin and ethylene crosstalk is well-established [69]. In total, 59 ethylene-related genes were found to be transcriptionally altered in response to auxin in the moso bamboo root (Fig. 5c, Additional file 17: Table S22). The application of auxin normally upregulates the transcription of genes encoding ethylene biosynthetic enzymes and leads to increased ethylene biosynthesis [69]. In accordance with this point, we found that 1 putative aminocyclopropane-1-carboxylic acid synthase (*ACS*) gene (*PH01000000G2120*), which catalyzes the rate-limiting step in ethylene synthesis, was upregulated under our exogenous auxin treatment condition. The second group of auxin-responsive ethylene genes is involved in a signaling pathway. Twenty-five *ERFs*, including one putative ethylene receptor gene (*PH01005673G0050*), were auxin responsive, and most of these genes were upregulated (Fig. 5c, Additional file 17: Table S22). In general, the enhanced expression of ethylene biosynthesis and signaling genes after auxin treatment showed the positive crosstalk between these two plant hormones.

The auxin signaling pathway not only controls various aspects of plant growth and development but also plays roles in plant environmental adaptation via crosstalk with some stress-related phytohormones, such as abscisic acid (ABA), jasmonate (JA) or salicylic acid (SA) [37]. Our results showed that 20 genes involved in the ABA-related pathways are auxin-responsive (Fig. 5d, Additional file 18: Table S23). Of these, *PH01000029G2070*, the putative ABA-responsive element binding protein 3 (*AREB3*) transcription factor in moso bamboo, was strongly induced by exogenous auxin treatment. We further identified 32 and 30 auxin-responsive genes that are involved in the JA and SA pathways, respectively (Fig. 5e and f, Additional file 19: Table S24 and Additional file 20: Table S25).

## Discussion

Bamboo is well known for its rapid growth and high level of woodiness. At the cellular level, the fast growth of the bamboo culm is mainly due to cell elongation and division [24]. Auxin is one of the well-known phytohormones that controls cell division and expansion [13]. However, the genes involved in auxin pathway and the global auxin-response profiling in moso bamboo still need to be investigated.

Here, we showed that exogenous NAA inhibit bamboo root growth (Fig. 1). We identified the key gene families involved in auxin biosynthesis (*PhYUC* family; 13 members), auxin transport (*PhPIN*, *PhPILS* and *PhLAX* families; 6, 8, and 7 members respectively), auxin receptors (*PhAFB* family; 10 members) and auxin signaling (*PhIAA* and *PhARF* families; 43 and 41 members, respectively) in moso bamboo (Fig. 2). Our analysis indicated the importance of compartmentalized auxin homeostasis and auxin signaling throughout the plant kingdom. Compared with Arabidopsis and rice, most of the auxin action- related gene families in moso bamboo have extensively expended their numbers of members (Additional file 2: Figure S4), suggesting that the auxin action in moso bamboo is more complicated and diverse.

Until now, no auxin-related gene in moso bamboo was functionally characterized. The phylogenetic relationships of the auxin pathway genes may suggest their putative roles in moso bamboo. For example, *OsYUC1* encodes the key enzyme contributing to IAA biosynthesis in rice [70], and it will be very interesting to determine the function of its ortholog in moso bamboo *PhYUC11* (Additional file 2: Figure S4). *PhPIN4* is most closely related to *OsPIN10a* and *OsPIN10b* (Additional file 2: Figure S4), which are supposed to be monocot-specific [45]. In Arabidopsis, *AtLAX3* is reported to promote the initiation of lateral root primordia by increasing the expression of a selection of cell-wall-remodeling enzymes [71]. In bamboo, we identified its closest ortholog *PhLAX6* (Additional file 2: Figure S4). Heterodimerization between Aux/IAA and ARF proteins are crucial for their unique biological functions in different tissues in Arabidopsis [22]. The studies of the protein-protein interactions and gene co-expression maps in Arabidopsis, together with our phylogenetic analysis (Additional file 2: Figure S4), will facilitate future studies of the interaction maps of PhIAAs and PhARFs. This analysis is a key step to understanding the auxin action network in moso bamboo.

Previous studies have shown that elevated cellular auxin levels stimulate the transcriptional changes of genes involved in auxin biosynthesis, conjugation, transport, and signaling [51]. We performed the KEGG analysis of the differentially expressed genes, and our results showed that those differentially expressed genes were classified into 12 pathways, and the genes involved in plant hormone signal transduction were predominantly enriched (33 DEGs) (Additional file 21: Table S26). Our results also showed that a number of auxin pathway genes changed their expression, which is consistent with a previous report (Fig. 4, Additional file 10: Table S15). While there are some exceptions, for example, all *AUX1/LAX* genes in Arabidopsis increased their transcripts after auxin treatment, but in moso bamboo, only 3 genes changed their expression levels: 2 *PhLAX* genes (*PhLAX4* and *PhLAX6*) were down-regulated, whereas the *YUC* genes involved in auxin biosynthesis (*PhYUC4*, *5*, *9* and *13*) were mostly upregulated (Fig. 4). The latter result is opposite to the results in Arabidopsis, in which the *YUC* genes are downregulated through the feedback pathways [72]. These results indicate that the mechanisms that control auxin concentrations in moso bamboo are more complicated, and the expression of related genes may tightly and precisely controlled by the properties of external stimuli, different tissues, or various developmental stages. Interestingly, *PhYUC3* is the only *YUC* gene that was downregulated by exogenous auxin in the moso bamboo root (Fig. 4), suggesting that PhYUC3 may encode one of the rate-limiting enzymes for auxin biosynthesis in the bamboo root. Further genetic and biochemical experiments are needed to characterize the potentially important function of these genes.

NAA, IAA and IBA are three most commonly used compounds in auxin research. Although they were transported in different ways in plant cells, they cause similar physiological response by changing overlapping downstream gene expressions [3, 73]. As a preliminary test on the effects of various auxins in moso bamboo, we treated the seedlings with 5 μM IAA or 5 μM IBA for 4 h (similar conditions as the NAA treatment we used), and randomly selected genes whose expressions were changed after NAA treatment for qRT-PCR analysis. The genes we checked include: Ph01003158G0110 and Ph01000099G0730 for GH3 family; Ph01000001G1450 and Ph01004534G0130 for SAUR family; Ph01001249G0310 for LBD family; Ph01000025G1600, Ph01000554G0550, Ph01003159G0070 and Ph01001154G0590 for AUX/IAA family. Our results showed that these genes had similar expression patterns after IAA or IBA treatments, while the extents of the changes were different (Additional file 2: Figure S10), these results support the previous findings that different auxins have both common and specific efficacies to activate various auxin signaling pathways [74]. Moreover, previous reports also showed that different auxins cause distinct but overlapping changes in gene expression, probably due to the differences in their metabolism, transport or interaction with the signaling components [75, 76]. Thereby, it is needed to perform more detailed and systematic studies to unveil the effects of various auxins on the expression of auxin-related genes in moso bamboo in the future.

We also identified the genes that were regulated by exogenous auxin (Fig. 5). Notably, our results showed that a significant number of auxin-responsive genes are involved in the biosynthesis, metabolism or signaling pathway of other phytohormone like cytokinin, ethylene, Gibberellin, ABA, SA and JA (Fig. 5). Various plant hormones affect similar cellular processes through complicated interactions. Our results provide insights into how auxin pathways crosstalk with other plant hormone pathways to regulate moso bamboo growth and development, which should be investigated in the future.

## Conclusion

In summary, we established a general overview of the main pathways involves in auxin synthesis, transport, receptor and signaling, and the global transcriptional profiling of auxin response in moso bamboo. The results from this study provided information for the elucidation of the possible functions of auxin action-related genes in bamboo. In the future, more experimental and bioinformatics work is needed to fully understand the functions of these important candidate genes and the regulatory mechanisms of some important auxin action-related proteins in this particular species.

## Additional files

Additional file 1: Table S1. List of putative PhYUC family genes in maso bamboo. **Table S2.** List of putative PhPIN/PILS genes in maso bamboo. **Table S3.** List of putative PhLAX genes in maso bamboo. **Table S4.** List of putative auxin binding factors (AFB) in bamboo. **Table S5.** List of putative PhAUX/IAA genes in maso bamboo. **Table S6.** List of putative PhARF genes in maso bamboo. **Table S7.** List of primers used for qPCR analysis. (DOCX 28 kb)

Additional file 2: Figure S1. General overview of auxin biosynthesis, transport, and signaling pathway. **Figure S2.** Multiple sequence alignment, conserved motif and gene structure analysis of members involved in auxin concentration. **Figure S3.** Multiple sequence alignment, conserved motif and gene structure analysis of members involved in auxin signaling. **Figure S4.** Phylogenetic analysis of families related to auxin action in moso bamboo, rice, and Arabidopsis. **Figure S5.** qRT-PCR analysis of auxin responsive marker genes in response to exogenous auxin treatment. **Figure S6.** Heat-map of Euclidean distance among the 6 bamboo RNA-Seq libraries used in this study. **Figure S7.** Validation of RNA-Seq results by qPCR. **Figure S8.** qRTPCR confirmation of the expressions of auxin-related genes. **Figure S9.** Tissue expression patterns of the auxin related genes. **Figure S10.** qRT-PCR analysis of auxin responsive marker genes in response to exogenous IAA and IBA treatment. (PDF 3914 kb)

Additional file 3: Table S8. List of the genes in the RNA-Seq analysis. (TXT 4491 kb)

Additional file 4: Table S9. Expression of PhYUC family members in response to auxin treatment. (TXT 1 kb)

Additional file 5: Table S10. Expression of PhPIN/PhPILS family members in response to auxin treatment. (TXT 1 kb)

Additional file 6: Table S11. Expression of PhLAX family members in response to auxin treatment. (TXT 1 kb)

Additional file 7: Table S12. Expression of PhAFB family members in response to auxin treatment. (TXT 1 kb)

Additional file 8: Table S13. Expression of PhIAA family members in response to auxin treatment. (TXT 5 kb)

Additional file 9: Table S14. Expression of PhARF family members in response to auxin treatment. (TXT 5 kb)

Additional file 10: Table S15. List of the auxin action related genes with expression change in response to exogenous auxin treatment. (TXT 5 kb)

Additional file 11: Table S16. Expression of putative PhGH3 members in response to auxin treatment. (TXT 824 bytes)

Additional file 12: Table S17. Expression of PhSAUR family members in response to auxin treatment. (TXT 1 kb)

Additional file 13: Table S18. Expression of putative PhLBD members in response to auxin treatment. (TXT 1 kb)

Additional file 14: Table S19. Expression changes of cell wall related genes in response to exogenous auxin treatment. (TXT 6 kb)

Additional file 15: Table S20. Expression changes of cytokinin related genes in response to exogenous auxin treatment. (TXT 1 kb)

Additional file 16: Table S21. Expression changes of GA related genes in response to exogenous auxin treatment. (TXT 1 kb)

Additional file 17: Table S22. Expression changes of ethylene related genes in response to exogenous auxin treatment. (TXT 7 kb)

Additional file 18: Table S23. Expression changes of ABA related genes in response to exogenous auxin treatment. (TXT 2 kb)

Additional file 19: Table S24. Expression changes of JA related genes in response to exogenous auxin treatment. (TXT 3 kb)

Additional file 20: Table S25. Expression changes of SA related genes in response to exogenous auxin treatment. (TXT 3 kb)

Additional file 21: Table S26. The statistical enrichment of DEGs in the KEGG pathways. (TXT 4 kb)

## Abbreviations

ABA: Abscisic acid
ACS: Aminocyclopropane-1-carboxylic acid synthase
AFB: Auxin signaling F-BOX protein
AREB: ABA-responsive element binding protein
ARFs: Auxin response factors
AUX/IAA: auxin/indole-3-aceticacid
AUX1/LAX: Auxin 1/like-AUX1
AuxRE: Auxin-responsive *cis*-element
BLAST: Basic local alignment search tool
CKX: Cytokinin oxidase/dehydrogenase
CTD domain: Carboxy-terminal dimerization domain
DBD: DNA binding domain
DEGs: Differentially expressed genes
dUTP: Deoxyuridine triphosphate
ER: Endoplasmic reticulum
ERFs: Ethylene responsive factors
FDR: False discovery rate
FPKM: Fragment per kilobase of transcript per million mapped reads
GA: Gibberellin acid
GH3: Gretchen Hagen 3
HD zip: Homeodomain zip
HMM: Hidden Markov model
IAA: Indole-3-acetic acid
IBA: Indole-3-butytric acid
IPA: Indole-3-pyruvate
JA: Jasmonic acid
LBD: Lateral organ boundaries domain
LOB: Lateral organ boundaries
MS: Murashige and skoog
NAA: Naphthaleneacetic acid
ORF: Open reading frame
PIN/PILS: PIN-FORMED/PIN-like
PMEs: Pectinmethylesterases
qRT-PCR: Quantiative real time PCR
RNA-Seq: RNA-Sequencing
SA: Salicylic acid
SAUR: Small auxin-up RNA
SE: Standard error
TAA: Tryptophan aminotransferase
TIR1: Transport inhibitor response 1
XTHs: Xyloglucan endotransglycosylases
YUC: YUCCA
