# Supplementary material for: Genome-wide analysis and transcriptomic profiling of the auxin biosynthesis, transport and signaling family genes in moso bamboo (Phyllostachys heterocycla)
Source: BMC Genomics. 2017 Nov 13;18:870. doi: 10.1186/s12864-017-4250-0 (PMC5683460; doi:10.1186/s12864-017-4250-0)
Supplement: Supplementary file 1 — List of putative PhYUC family genes in maso bamboo. Table S2. List of putative PhPIN/PILS genes in maso bamboo. Table S3. List of putative PhLAX genes in maso bamboo. Table S4. List of putative auxin binding factors (AFB) in bamboo. Table S5. List of putative PhAUX/IAA genes in maso bamboo. Table S6. List of putative PhARF genes in maso bamboo. Table S7. List of primers used for qPCR analysis. (DOCX 28 kb) [file 12864_2017_4250_MOESM1_ESM.docx]

| **Table S1. List of putative PhYUC family genes in maso bamboo** | | | | | | | | |
| --- | --- | --- | --- | --- | --- | --- | --- | --- |
| **Gene_name** | **Gene ID** | **Exon** | **Intron** | **ORF length** | **AA length** | **AA MW** | **Domain** | **putative subcellular localization** |
| PhYUC1 | PH01000006G2190 | 5 | 4 | 1383 | 460 | 49752.2 | NADB_Rossmann superfamily | Cytoplasm. |
| PhYUC2 | PH01000094G0340 | 2 | 1 | 774 | 257 | 28060.4 | NADB_Rossmann superfamily | Chloroplast. Cytoplasm. Nucleus. |
| PhYUC3 | PH01000209G0350 | 6 | 5 | 1131 | 376 | 41661.6 | NADB_Rossmann superfamily UBN2 superfamily | Cytoplasm. |
| PhYUC4 | PH01000209G0370 | 2 | 1 | 861 | 286 | 31611 | NADB_Rossmann superfamily | Mitochondrion. |
| PhYUC5 | PH01000209G0380 | 3 | 2 | 513 | 170 | 18798.4 | FMO-like | Chloroplast. |
| PhYUC6 | PH01000262G0890 | 5 | 4 | 1575 | 524 | 58841.5 | NADB_Rossmann superfamily FMO-like | Chloroplast. |
| PhYUC7 | PH01000262G0940 | 5 | 4 | 1560 | 519 | 57721 | NADB_Rossmann superfamily Pyr_redox_3 | Chloroplast. Cytoplasm |
| PhYUC8 | PH01000875G0030 | 2 | 1 | 1248 | 415 | 45992 | NADB_Rossmann superfamily | Chloroplast |
| PhYUC9 | PH01002317G0180 | 4 | 3 | 1074 | 357 | 39366.6 | NADB_Rossmann superfamily | No indication |
| PhYUC10 | PH01002560G0100 | 4 | 3 | 1212 | 403 | 43700.3 | NADB_Rossmann superfamily | Chloroplast. Mitochondrion. |
| PhYUC11 | PH01002882G0160 | 2 | 1 | 261 | 86 | 9489.4 | PRKO6292 | Chloroplast. Golgi apparatus |
| PhYUC12 | PH01003231G0010 | 4 | 3 | 1659 | 552 | 170603.5 | NADB_Rossmann superfamily FMO-like | Chloroplast. |
| PhYUC13 | PH01003290G0170 | 3 | 2 | 597 | 198 | 21126.1 | NADB_Rossmann superfamily | Chloroplast. Cytoplasm. Mitochondrion. |
| **Table S2. List of putative PhPIN/PILS genes in maso bamboo** | | | | | | | | |
| **Gene_name** | **Gene ID** | **Exon** | **Intron** | **ORF length** | **AA length** | **AA MW** | **Domain** | **putative subcellular localization** |
| PhPIN1 | PH01000051G0950 | 7 | 6 | 1836 | 611 | 65100.3 | Mem_trans superfamily | Cell membrane. |
| PhPIN2 | PH01000065G0400 | 7 | 6 | 1818 | 605 | 66295.7 | Mem_trans superfamily | Cytoplasm. |
| PhPIN3 | PH01000484G0740 | 6 | 5 | 1785 | 594 | 64920.5 | Mem_trans superfamily | Cytoplasm. |
| PhPIN4 | PH01000498G0530 | 5 | 4 | 1644 | 547 | 59206.8 | Mem_trans superfamily | Cell membrane. Chloroplast. |
| PhPIN5 | PH01000717G0260 | 7 | 6 | 1413 | 470 | 51058.8 | Mem_trans superfamily | Cytoplasm. |
| PhPIN6 | PH01003160G0100 | 7 | 6 | 999 | 332 | 36275.6 | Mem_trans superfamily Bzip superfamily | Chloroplast. Nucleus. Peroxisome. |
| PhPILS1 | PH01000009G1330 | 10 | 9 | 1296 | 431 | 46502.7 | Mem_trans superfamily | Cell membrane. |
| PhPILS2 | PH01000070G0050 | 9 | 8 | 1068 | 355 | 37984.3 | Mem_trans superfamily | Cell membrane. Golgi apparatus. Vacuole. |
| PhPILS3 | PH01000112G0430 | 10 | 9 | 1293 | 430 | 46346.6 | Mem_trans superfamily | Cell membrane. |
| PhPILS4 | PH01000261G0540 | 10 | 9 | 1275 | 424 | 45772.2 | Mem_trans superfamily | Cell membrane. |
| PhPILS5 | PH01000642G0520 | 8 | 7 | 1260 | 419 | 45143.6 | Mem_trans superfamily | Cell membrane. |
| PhPILS6 | PH01000813G0450 | 1 | 0 | 1356 | 451 | 49356.6 | Mem_trans superfamily | Cell membrane. |
| PhPILS7 | PH01001573G0200 | 9 | 8 | 1023 | 340 | 36088.4 | Mem_trans superfamily | Cell membrane. Golgi apparatus. |
| PhPILS8 | PH01002616G0280 | 8 | 7 | 1278 | 425 | 46170.3 | Mem_trans superfamily | Cell membrane |
|  |  |  |  |  |  |  |  |  |
| **Table S3. List of putative PhLAX genes in maso bamboo** | | | | | | | | |
| **Gene_name** | **Gene ID** | **Exon** | **Intron** | **ORF length** | **AA length** | **AA MW** | **Domain** | **putative subcellular localization** |
| PhLAX1 | PH01000004G3230 | 7 | 6 | 1434 | 477 | 53145.7 | SLC5-0-like_sbd superfamily | Cell membrane |
| PhLAX2 | PH01000090G0510 | 7 | 6 | 1482 | 493 | 54837.4 | SLC5-1-like_sbd superfamily | Cell membrane. |
| PhLAX3 | PH01000339G0320 | 7 | 6 | 1593 | 530 | 58721.7 | SLC5-2-like_sbd superfamily | Cell membrane. |
| PhLAX4 | PH01000373G0290 | 8 | 7 | 1539 | 512 | 56747.7 | SLC5-3-like_sbd superfamily | Cell membrane. |
| PhLAX5 | PH01000413G0700 | 7 | 6 | 1575 | 524 | 58195.1 | SLC5-4-like_sbd superfamily | Cell membrane. |
| PhLAX6 | PH01001222G0340 | 6 | 5 | 1431 | 476 | 53305.8 | SLC5-5-like_sbd superfamily | Cell membrane. |
| PhLAX7 | PH01003226G0090 | 8 | 7 | 1440 | 479 | 53258.3 | SLC5-6-like_sbd superfamily | Cell membrane. |
|  |  |  |  |  |  |  |  |  |
| **Table S4. List of putative auxin binding factors (AFB) in bamboo** | | | | | | | | |
| **Gene_name** | **Gene ID** | **Exon** | **Intron** | **ORF length** | **AA length** | **AA MW** | **Domain** | **putative subcellular localization** |
| PhAFB1 | PH01000016G0700 | 3 | 2 | 1728 | 575 | 64307.52 | F-box AMN1 LRR_RT | Nucleus |
| PhAFB2 | PH01000035G0470 | 4 | 3 | 1854 | 617 | 6762.65 | AMN1 | Nucleus |
| PhAFB3 | PH01000038G1830 | 4 | 3 | 1731 | 576 | 63109.05 | AMN1 LRR_RT | Nucleus |
| PhAFB4 | PH01000080G0540 | 3 | 2 | 1857 | 618 | 68391.39 | F-box AMN1 | Nucleus |
| PhAFB5 | PH01000115G1470 | 4 | 3 | 1632 | 543 | 59938.2 | AMN1 | Nucleus |
| PhAFB6 | PH01000173G0090 | 3 | 2 | 1881 | 626 | 69121.47 | AMN1 | Nucleus |
| PhAFB7 | PH01000245G0260 | 3 | 2 | 1866 | 621 | 68618.43 | AMN1 | Nucleus |
| PhAFB8 | PH01000899G0100 | 4 | 3 | 1851 | 616 | 67293.75 | AMN1 LRR_RT | Nucleus |
| PhAFB9 | PH01000943G0520 | 3 | 2 | 1863 | 620 | 68267.1 | AMN1 | Nucleus |
| PhAFB10 | PH01001592G0190 | 4 | 3 | 1779 | 592 | 64919.22 | AMN1 LRR_RT | Nucleus |
| **Table S5. List of putative PhAUX/IAA genes in maso bamboo** | | | | | | | | |
| **Gene_name** | **Gene ID** | **Exon** | **Intron** | **ORF length** | **AA length** | **AA MW** | **Domain** | **putative subcellular localization** |
| PhIAA1 | PH01000003G3650 | 5 | 4 | 576 | 191 | 20200.51 | AUX_IAA | Nucleus. |
| PhIAA2 | PH01000025G1600 | 3 | 2 | 705 | 234 | 24325.08 | AUX_IAA | Nucleus. |
| PhIAA3 | PH01000025G1630 | 4 | 3 | 573 | 190 | 20213.23 | AUX_IAA | Nucleus. |
| PhIAA4 | PH01000075G0200 | 3 | 2 | 582 | 193 | 20312.21 | AUX_IAA | Nucleus. |
| PhIAA5 | PH01000105G1710 | 3 | 2 | 537 | 178 | 19256.73 | AUX_IAA | Nucleus. |
| PhIAA6 | PH01000116G0910 | 15 | 14 | 2454 | 817 | 90054.90 | Auxin_resp AUX_IAA BfiI_C_EcoRII | Nucleus. |
| PhIAA7 | PH01000212G0710 | 7 | 6 | 615 | 204 | 22180.09 | AUX_IAA | Nucleus. |
| PhIAA8 | PH01000214G1340 | 3 | 2 | 1035 | 341 | 37475.26 | AUX_IAA | Nucleus. |
| PhIAA9 | PH01000222G0180 | 15 | 14 | 2253 | 750 | 82952.31 | BfiI_C_EcoRII Auxin_resp AUX_IAA FBOX | Nucleus. |
| PhIAA10 | PH01000356G1070 | 18 | 17 | 906 | 301 | 32507.45 | AUX_IAA | Nucleus. |
| PhIAA11 | PH01000384G0170 | 4 | 3 | 2292 | 763 | 84747.78 | BfiI_C_EcoRII Auxin_resp AUX_IAA | Nucleus. |
| PhIAA12 | PH01000429G1070 | 6 | 5 | 1221 | 406 | 44497.01 | AUX_IAA | Nucleus. |
| PhIAA13 | PH01000497G0760 | 3 | 2 | 621 | 206 | 22185.06 | AUX_IAA | Nucleus. |
| PhIAA14 | PH01000554G0550 | 3 | 2 | 651 | 216 | 23309.73 | AUX_IAA | Nucleus. |
| PhIAA15 | PH01000592G0620 | 3 | 2 | 618 | 205 | 22206.16 | AUX_IAA | Nucleus. |
| PhIAA16 | PH01000626G0040 | 14 | 13 | 2082 | 693 | 77499.65 | BfiI_C_EcoRII Auxin_resp AUX_IAA | Nucleus. |
| PhIAA17 | PH01000627G0150 | 6 | 5 | 1023 | 340 | 36921.00 | AUX_IAA | Nucleus. |
| PhIAA18 | PH01000676G0500 | 4 | 3 | 711 | 236 | 25215.71 | AUX_IAA | Nucleus. |
| PhIAA19 | PH01000812G0170 | 4 | 3 | 252 | 83 | 9190.42 | AUX_IAA | Nucleus. |
| PhIAA20 | PH01001098G0440 | 2 | 1 | 459 | 152 | 16017.95 | AUX_IAA | Nucleus. |
| PhIAA21 | PH01001119G0310 | 3 | 2 | 522 | 173 | 18313.10 | AUX_IAA | Nucleus. |
| PhIAA22 | PH01001154G0590 | 4 | 3 | 735 | 244 | 25631.48 | AUX_IAA | Nucleus. |
| PhIAA23 | PH01001175G0280 | 3 | 2 | 591 | 196 | 21334.65 | AUX_IAA | Nucleus. |
| PhIAA24 | PH01001446G0210 | 3 | 2 | 555 | 184 | 19718.72 | AUX_IAA | Nucleus. |
| PhIAA25 | PH01001567G0470 | 3 | 2 | 810 | 269 | 28450.11 | AUX_IAA | Nucleus. |
| PhIAA26 | PH01001580G0170 | 6 | 5 | 957 | 318 | 34168.88 | AUX_IAA | Nucleus. |
| PhIAA27 | PH01001669G0110 | 2 | 1 | 816 | 271 | 28643.34 | AUX_IAA | Nucleus. |
| PhIAA28 | PH01001671G0190 | 3 | 2 | 420 | 139 | 15047.62 | AUX_IAA | Nucleus. |
| PhIAA29 | PH01001792G0060 | 5 | 4 | 828 | 275 | 29710.21 | AUX_IAA | Nucleus. |
| PhIAA30 | PH01002806G0200 | 14 | 13 | 2523 | 840 | 92950.54 | BfiI_C_EcoRII Auxin_resp AUX_IAA | Nucleus. |
| PhIAA31 | PH01003159G0070 | 4 | 3 | 792 | 263 | 28229.01 | AUX_IAA | Nucleus. |
| PhIAA32 | PH01003159G0100 | 3 | 2 | 669 | 222 | 23832.69 | AUX_IAA | Nucleus. |
| PhIAA33 | PH01003333G0020 | 3 | 2 | 531 | 176 | 19744.92 | AUX_IAA | Nucleus. |
| PhIAA34 | PH01003595G0090 | 5 | 4 | 720 | 239 | 25820.17 | AUX_IAA | Nucleus. |
| PhIAA35 | PH01003631G0130 | 13 | 12 | 2499 | 832 | 92637.23 | BfiI_C_EcoRII Auxin_resp AUX_IAA | Nucleus. |
| PhIAA36 | PH01004159G0030 | 14 | 13 | 1527 | 508 | 53540.11 | AUX_IAA Ntn_hydrolase superfamily | Nucleus. |
| PhIAA37 | PH01004448G0020 | 6 | 5 | 942 | 313 | 34347.08 | AUX_IAA | Nucleus. |
| PhIAA38 | PH01005193G0070 | 4 | 3 | 816 | 271 | 28912.55 | AUX_IAA | Nucleus. |
| PhIAA39 | PH01005322G0010 | 14 | 13 | 2379 | 792 | 87429.78 | BfiI_C_EcoRII Auxin_resp AUX_IAA | Nucleus. |
| PhIAA40 | PH01005434G0050 | 5 | 4 | 771 | 256 | 27438.83 | AUX_IAA | Nucleus. |
| PhIAA41 | PH01005757G0030 | 6 | 5 | 771 | 256 | 27621.87 | AUX_IAA | Nucleus. |
| PhIAA42 | PH01007612G0010 | 14 | 13 | 2019 | 672 | 74717.68 | BfiI_C_EcoRII Auxin_resp AUX_IAA | Nucleus. |
| PhIAA43 | PH01014988G0010 | 3 | 2 | 564 | 188 | 20119.95 | AUX_IAA | Nucleus. |
| **Table S6. List of putative PhARF genes in maso bamboo** | | | | | | | | |
| **Gene_name** | **Gene ID** | **Exon** | **Intron** | **ORF length** | **AA length** | **AA MW** | **Domain** | **putative subcellular localization** |
| PhARF1 | PH01000002G3110 | 17 | 16 | 2487 | 828 | 92494.24 | B3-DNA binding Auxin-resp CTD | Nucleus |
| PhARF2 | PH01000011G0660 | 12 | 11 | 1665 | 554 | 61313.36 | B3-DNA binding Auxin-resp | Nucleus |
| PhARF3 | PH01000014G0610 | 11 | 10 | 1284 | 427 | 47285.9 | B3-DNA binding CTD | Nucleus |
| PhARF4 | PH01000018G0940 | 12 | 11 | 2034 | 677 | 74314.65 | B3-DNA binding Auxin-resp | Nucleus |
| PhARF5 | PH01000044G0540 | 3 | 2 | 1320 | 439 | 47422.84 | B3-DNA binding Auxin-resp CTD | Nucleus |
| PhARF6 | PH01000046G0220 | 11 | 10 | 2205 | 734 | 80456.86 | B3-DNA binding Auxin-resp | Nucleus |
| PhARF7 | PH01000057G1420 | 13 | 12 | 2787 | 928 | 102860 | B3-DNA binding Auxin-resp CTD | Nucleus |
| PhARF8 | PH01000087G1340 | 12 | 11 | 2217 | 738 | 82040.04 | B4-DNA binding Auxin-resp CTD | Nucleus |
| PhARF9 | PH01000093G0670 | 9 | 8 | 2253 | 750 | 83224.64 | B5-DNA binding Auxin-resp CTD | Nucleus |
| PhARF10 | PH01000114G0050 | 15 | 14 | 3774 | 1257 | 138978.62 | B6-DNA binding Auxin-resp CTD | Nucleus |
| PhARF11 | PH01000116G0910 | 15 | 14 | 2454 | 817 | 90054.9 | B7-DNA binding Auxin-resp CTD | Nucleus |
| PhARF12 | PH01000176G0540 | 14 | 13 | 3366 | 1121 | 124655.68 | B8-DNA binding Auxin-resp CTD | Nucleus |
| PhARF13 | PH01000183G0570 | 14 | 13 | 2694 | 897 | 99047.28 | B9-DNA binding Auxin-resp CTD | Nucleus |
| PhARF14 | PH01000222G0180 | 15 | 14 | 2253 | 750 | 82952.31 | B10-DNA binding Auxin-resp CTD | Nucleus |
| PhARF15 | PH01000227G0020 | 15 | 14 | 2727 | 908 | 100656.02 | B11-DNA binding Auxin-resp CTD | Nucleus |
| PhARF16 | PH01000237G0420 | 7 | 6 | 1545 | 514 | 56406.77 | B12-DNA binding Auxin-resp | Nucleus |
| PhARF17 | PH01000259G1120 | 9 | 8 | 2715 | 904 | 100651.41 | B13-DNA binding Auxin-resp CTD | Nucleus |
| PhARF18 | PH01000277G0820 | 10 | 9 | 1776 | 591 | 64646.15 | B14-DNA binding Auxin-resp | Nucleus |
| PhARF19 | PH01000305G0690 | 3 | 2 | 1908 | 635 | 69420.06 | B15-DNA binding Auxin-resp | Nucleus |
| PhARF20 | PH01000384G0170 | 18 | 17 | 2292 | 763 | 84747.78 | B13-DNA binding Auxin-resp CTD | Nucleus |
| PhARF21 | PH01000483G0220 | 13 | 12 | 2601 | 866 | 96094.52 | B14-DNA binding Auxin-resp CTD | Nucleus |
| PhARF22 | PH01000548G0300 | 11 | 10 | 2958 | 985 | 110042.99 | B15-DNA binding Auxin-resp CTD | Nucleus |
| PhARF23 | PH01000623G0440 | 14 | 13 | 3561 | 1186 | 131927.52 | B16-DNA binding Auxin-resp CTD | Nucleus |
| PhARF24 | PH01000626G0040 | 14 | 13 | 2082 | 693 | 77499.65 | B17-DNA binding Auxin-resp CTD | Nucleus |
| PhARF25 | PH01000667G0020 | 13 | 12 | 1914 | 637 | 71429.59 | B18-DNA binding Auxin-resp | Nucleus |
| PhARF26 | PH01000845G0410 | 7 | 6 | 4533 | 1510 | 166237.37 | B19-DNA binding Auxin-resp | Nucleus |
| PhARF27 | PH01001026G0300 | 5 | 4 | 2235 | 744 | 81013.69 | B17-DNA binding Auxin-resp CTD | Nucleus |
| PhARF28 | PH01001285G0430 | 5 | 4 | 1812 | 603 | 65328.51 | B19-DNA binding Auxin-resp | Nucleus |
| PhARF29 | PH01001555G0390 | 14 | 13 | 2637 | 878 | 97749.56 | B20-DNA binding Auxin-resp CTD | Nucleus |
| PhARF30 | PH01001690G0310 | 12 | 11 | 2106 | 701 | 76946.00 | B21-DNA binding Auxin-resp | Nucleus |
| PhARF31 | PH01001899G0250 | 11 | 10 | 2187 | 728 | 80828.69 | B22-DNA binding Auxin-resp CTD | Nucleus |
| PhARF32 | PH01002160G0180 | 13 | 12 | 2274 | 757 | 84589.23 | B23-DNA binding Auxin-resp CTD | Nucleus |
| PhARF33 | PH01002498G0280 | 2 | 1 | 1977 | 658 | 71994.19 | B24-DNA binding Auxin-resp | Nucleus |
| PhARF34 | PH01002685G0120 | 4 | 3 | 1818 | 605 | 65847.89 | B25-DNA binding Auxin-resp | Nucleus |
| PhARF35 | PH01002806G0200 | 14 | 13 | 2523 | 840 | 92950.54 | B26-DNA binding Auxin-resp CTD | Nucleus |
| PhARF36 | PH01002850G0020 | 13 | 12 | 1431 | 476 | 54281.92 | B27-DNA binding Auxin-resp | Nucleus |
| PhARF37 | PH01002857G0060 | 15 | 14 | 2127 | 708 | 78911.16 | B28-DNA binding Auxin-resp CTD | Nucleus |
| PhARF38 | PH01003096G0200 | 13 | 12 | 2766 | 921 | 102220.68 | B29-DNA binding Auxin-resp CTD | Nucleus |
| PhARF39 | PH01003631G0130 | 13 | 12 | 2499 | 832 | 92637.23 | B30-DNA binding Auxin-resp CTD | Nucleus |
| PhARF40 | PH01005322G0010 | 14 | 13 | 2379 | 792 | 87429.78 | B31-DNA binding Auxin-resp CTD | Nucleus |
| PhARF41 | PH01007612G0010 | 14 | 13 | 2019 | 672 | 74717.68 | B32-DNA binding Auxin-resp CTD | Nucleus |

| **Table S7 List of primers used for qPCR analysis** | | |  |
| --- | --- | --- | --- |
| **Name** | **Sequence** | **Usage** |  |
| PH01001945G0320-R | GGGATGTCTGGTTCGGCTTC | Used for QPCR |  |
| PH01001945G0320-F | TGGGAGGTCGTAGTGTCGCTT | Used for QPCR |  |
| PH01003538G0010-R | ACTACAAGAGGGTGTCGTGCCA | Used for QPCR |  |
| PH01003538G0010-F | GCTCATCTCAATCCAGTTTGTGTTC | Used for QPCR |  |
| PH01000788G0630-R | CTCTGCCGCAACCACCAAC | Used for QPCR |  |
| PH01000788G0630-F | GGGCGTCCAGTCGGCTAAT | Used for QPCR |  |
| PH01000900G0600-R | CGGGTGAAGTGGAAGGGGT | Used for QPCR |  |
| PH01000900G0600-F | GGAGAAGGGGACGGAGGTG | Used for QPCR |  |
| PH01001206G0640-R | AGATTCAGTGCCCCAAGCCT | Used for QPCR |  |
| PH01001206G0640-F | AGCAGATGGTGATGACGGCA | Used for QPCR |  |
| PH01000615G0020-R | CAGTCGGTGTCCATCAAGGG | Used for QPCR |  |
| PH01000615G0020-F | CGTTGAGGAAGGTCAGCGTC | Used for QPCR |  |
| PH01000592G0620-R | CCGCCAAGTTCGTGAAGGTT | Used for QPCR |  |
| PH01000592G0620-F | CGGATGGTGAAGTGGGAGAAG | Used for QPCR |  |
| PH01001175G0280-R | ACTCTCCTCCCTCCCCCAAG | Used for QPCR |  |
| PH01001175G0280-F | GGACGGTGAAGTGGGAGAAGA | Used for QPCR |  |
| PH01001567G0470-R | GAGGAGAAGGGGATGGGC | Used for QPCR |  |
| PH01001567G0470-F | GCATCCTGCCTCGCTTCTAC | Used for QPCR |  |
| PH01000114G0050-R | TCTCTGTGCCTCGCCGTTAT | Used for QPCR |  |
| PH01000114G0050-F | AATAGCCTCTTGCCACTCACG | Used for QPCR |  |
| PH01000176G0540-R | TGCTAACCGACAACCGACTAACA | Used for QPCR |  |
| PH01000176G0540-F | TGCCAGTTATTGTACCCATGTACCT | Used for QPCR |  |
| PH01003158G0110-R | GGGCACTACATCATCTACTGGGA | Used for QPCR |  |
| PH01003158G0110-F | CGTGTTCAGGGCGTTCTCC | Used for QPCR |  |
| PH01000099G0730-R | CCGCTCCAACATCATCTCCTC | Used for QPCR |  |
| PH01000099G0730-F | CTCGTCTTCTATCGTCGGCATTA | Used for QPCR |  |
| PH01000788G0760-R: | CTGTGGCGTCCTAGCTTCC | Used for QPCR |  |
| PH01000788G0760-F: | TATGGCTGTGTCTCGCACAT | Used for QPCR |  |
| PH01000757G0560-R: | CCGAAGTAGCATTCCGACGA | Used for QPCR |  |
| PH01000757G0560-F: | CATCATCACCCGCATTTGGC | Used for QPCR |  |
| PH01004534G0130-R: | CAAACTGCAAGTTGTTGGTGC | Used for QPCR |  |
| PH01004534G0130-F: | TTGGGTTCACGAGTGATGGC | Used for QPCR |  |
| PH01001363G0470-R: | TGTTCGTCGACAACGGGAG | Used for QPCR |  |
| PH01001363G0470-F: | ACATCTTCGCGCTCCAACAG | Used for QPCR |  |
| PH01000001G1450-R: | TTTGTGCCATCACGCCTACT | Used for QPCR |  |
| PH01000001G1450-F: | GGTGGGATGGGAATGGTACG | Used for QPCR |  |
| PH01001249G0310-R: | GATGTCGTACGCCCTGAGC | Used for QPCR |  |
| PH01001249G0310-F: | ATCGTCAACCCGATCTACGG | Used for QPCR |  |
| PhYUC5-R: | CCGTCGTCACCGATCAGTTT | Used for QPCR |  |
| PhYUC5-F: | CAAGATCAAGAGCGGCCAGA | Used for QPCR |  |
| PhYUC13-R: | GACGACGCGCTGTTGTATTC | Used for QPCR |  |
| PhYUC13-F: | GGGGAGTGACTTCTTCACCG | Used for QPCR |  |
| PhPILS5-R: | CCTCTTCATCACGCCGTAGG | Used for QPCR |  |
| PhPILS5-F: | CTGCAAACTTCGGCAACCTG | Used for QPCR |  |
| PhPILS8-R: | GTACAATGGGTCGTGAGGCA | Used for QPCR |  |
| PhPILS8-F: | AACCTGACACAAGGGCTACG | Used for QPCR |  |
| PhLAX3-R: | TGGAACAGTATGCCGCTGAG | Used for QPCR |  |
| PhLAX3-F: | GTGACGCCAACACCGTAAAG | Used for QPCR |  |
| PhLAX4-R: | GGTGGAGTTGAACGGCATTG | Used for QPCR |  |
| PhLAX4-F: | GCATAACCAACTTCGTGCGG | Used for QPCR |  |
| PhIAA2-R: | TCCTCATAGGTCGGCACGTA | Used for QPCR |  |
| PhIAA2-F: | CTCCAAGGCGCTTGAGAAGA | Used for QPCR |  |
| PhIAA4-R: | AGTCCACGAGCTTATTCCCC | Used for QPCR |  |
| PhIAA4-F: | CAAACTCGTGAAGGTGGCCG | Used for QPCR |  |
| PhIAA13-R: | GTTCAGCACCTTCACCACAAC | Used for QPCR |  |
| PhIAA13-F: | CCGCAGGAATATCATGACCGT | Used for QPCR |  |
| PhIAA14-R: | GTTGTGGTGAAGGTGCTGAAC | Used for QPCR |  |
| PhIAA14-F: | GACCGTTCAGTCGGTGAAGAA | Used for QPCR |  |
| PhIAA15-R: | CACGTACTCCGTCCCGTTC | Used for QPCR |  |
| PhIAA15-F: | TTCGTGAAGGTTGCCGTGG | Used for QPCR |  |
| PhIAA22-R: | CATTGCCCTTGGTGTCCCAT | Used for QPCR |  |
| PhIAA22-F: | GTACCTGCGCAAGGAAACTG | Used for QPCR |  |
| PhIAA23-R: | TCCATCTCGTCGTTCCCAAC | Used for QPCR |  |
| PhIAA23-F: | GGCCAAATTCGTCAAGGTGG | Used for QPCR |  |
| PhIAA31-R: | CCTTGGCGCAAAGTCAATGG | Used for QPCR |  |
| PhIAA31-F: | ATGGATCTGCTCAACGGGTC | Used for QPCR |  |
| PhIAA38-R: | CCTTGTCCTCGTATGTCGGC | Used for QPCR |  |
| PhIAA38-F: | CCAGGATTTGTCCAGGGCAC | Used for QPCR |  |
| PhARF2-R: | AAGGTGCCTCTTCGGTTGAC | Used for QPCR |  |
| PhARF2-F: | ATTCTCAGTGCCACGTCGTT | Used for QPCR |  |
| PhARF3-R: | TGGGTACTTGTGTCACTCGC | Used for QPCR |  |
| PhARF3-F: | CAAGCTTCTGAGCCTCACCT | Used for QPCR |  |
| PhARF9-R: | AACTGAACCCACTGACGCAA | Used for QPCR |  |
| PhARF9-F: | TACTTTCGCTGCAGGAGGTG | Used for QPCR |  |
| PhARF10-R: | ATAGCCTCTTGCCACTCACG | Used for QPCR |  |
| PhARF10-F: | CCTCGCCGTTATGCAGAGAA | Used for QPCR |  |
| PhARF12-R: | GCATATCTCCTCACCGAGCC | Used for QPCR |  |
| PhARF12-F: | AAAGGCGGAACAGGGTTTCA | Used for QPCR |  |
| PhUBQ-R | TCAACCAAACCACTGTACCTCAG | Used for QPCR |  |
| PhUBQ-F | GGGTCGTCCAGTGTCCTCTATTA | Used for QPCR |  |
