# Supplementary material for: Genome-wide analysis and transcriptomic profiling of the auxin biosynthesis, transport and signaling family genes in moso bamboo (Phyllostachys heterocycla)
Source: BMC Genomics. 2017 Nov 13;18:870. doi: 10.1186/s12864-017-4250-0 (PMC5683460; doi:10.1186/s12864-017-4250-0)
Supplement: Supplementary file 2 — General overview of auxin biosynthesis, transport, and signaling pathway. Figure S2. Multiple sequence alignment, conserved motif and gene structure analysis of members involved in auxin concentration. Figure S3. Multiple sequence alignment, conserved motif and gene structure analysis of members involved in auxin signaling. Figure S4. Phylogenetic analysis of families related to auxin action in moso bamboo, rice, and Arabidopsis. Figure S5. qRT-PCR analysis of auxin responsive marker genes in response to exogenous auxin treatment. Figure S6. Heat-map of Euclidean distance among the 6 bamboo RNA-Seq libraries used in this study. Figure S7. Validation of RNA-Seq results by qPCR. Figure S8. qRTPCR confirmation of the expressions of auxin-related genes. Figure S9. Tissue expression patterns of the auxin related genes. Figure S10. qRT-PCR analysis of auxin responsive marker genes in response to exogenous IAA and IBA treatment. (PDF 3914 kb) [file 12864_2017_4250_MOESM2_ESM.pdf]

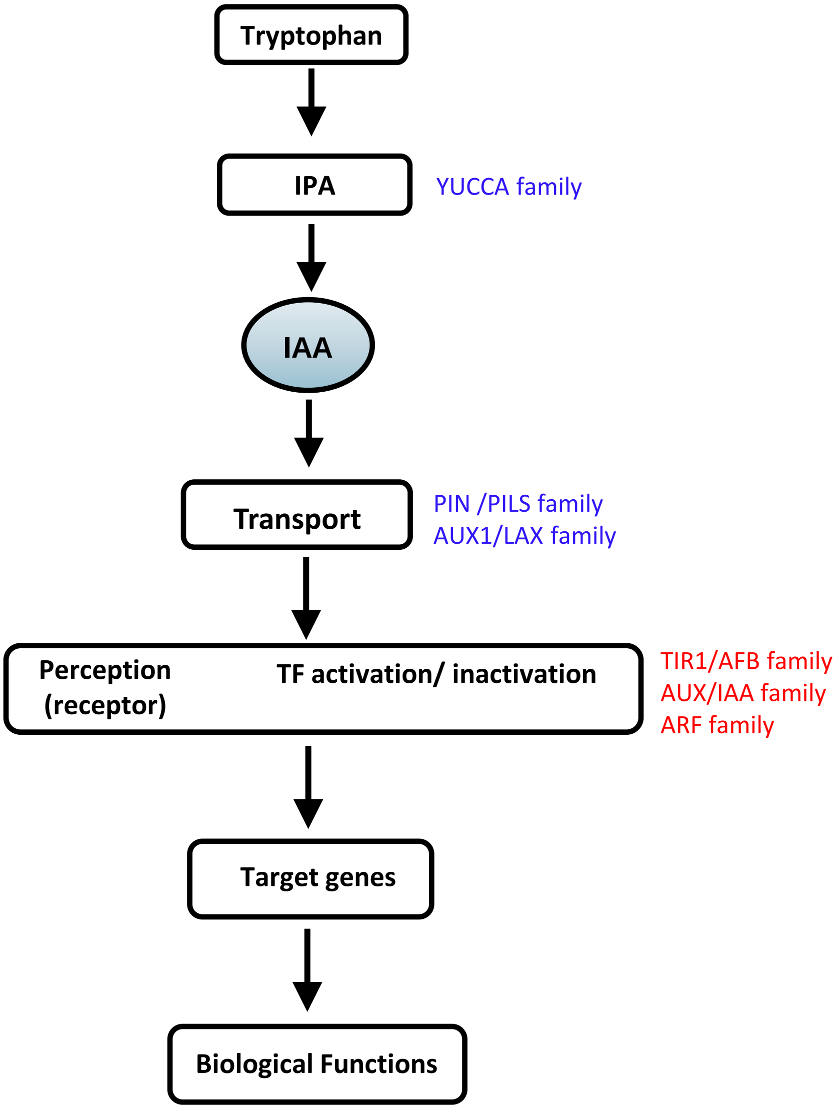
Tryptophan
IPA
YUCCA family
IAA
Transport
PIN /PILS family
AUX1/LAX family
Perception
(receptor)
TF activation/ inactivation
TIR1/AFB family
AUX/IAA family
ARF family
Target genes
Biological Functions

Figure S1. General overview of auxin biosynthesis, transport, and signaling pathway. Auxin is mainly synthesized through Tryptophan-dependent pathway and then transported by auxin transporters. The TIR1/AFB-AUX/IAA-ARF module plays the key role in auxin signaling pathway and induces the auxin response. In this paper, key families involved in auxin concentration (Blue color), namely the YUC family for auxin biosynthesis, the PIN/PILS family for auxin efflux transport, the AUX1/LAX family for auxin influx transport, and families related to the auxin signaling pathway (Red color), including TIR1/AFB family for auxin perception and the AUX/IAA and ARF families for signaling, were analyzed in moso bamboo.

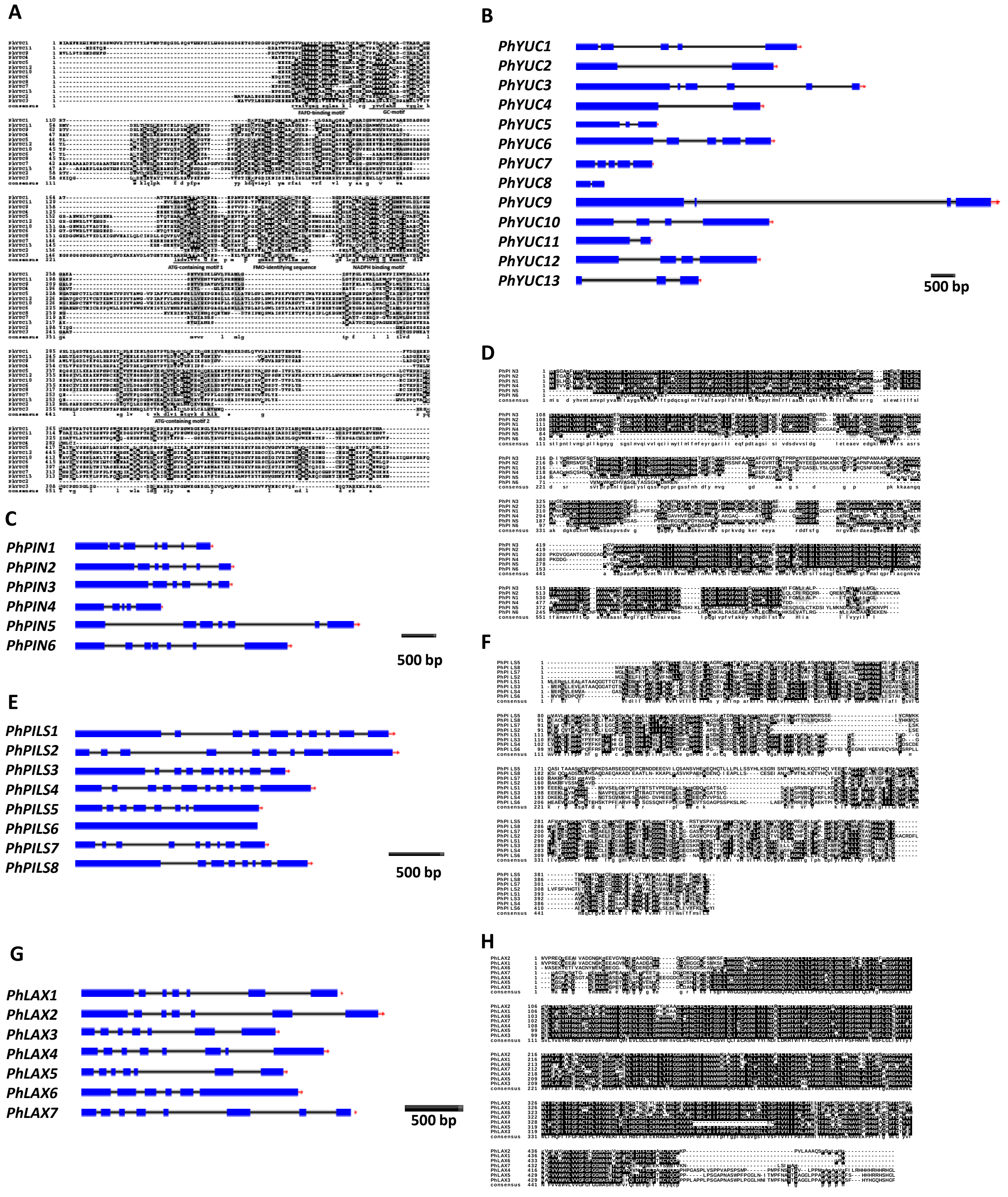
A
B
PhYUC1
PhYUC2
PhYUC3
PhYUC4
PhYUC5
PhYUC6
PhYUC7
PhYUC8
PhYUC9
PhYUC10
PhYUC11
PhYUC12
PhYUC13
500 bp
C
PhPIN1
PhPIN2
PhPIN3
PhPIN4
PhPIN5
PhPIN6
500 bp
D
E
PhPILS1
PhPILS2
PhPILS3
PhPILS4
PhPILS5
PhPILS6
PhPILS7
PhPILS8
500 bp
F
G
PhLAX1
PhLAX2
PhLAX3
PhLAX4
PhLAX5
PhLAX6
PhLAX7
500 bp
H

Figure S2. Multiple sequence alignment, conserved motif and gene structure analysis of members involved in auxin concentration. **A.** Schematic of amino acids conservation among PhYUC proteins. **B.** Gene structures of the *PhYUC* genes. **C.** Gene structures of the *PhPIN* members. **D.** Schematic of amino acids conservation among the moso bamboo *PhPIN.* **E.** Gene structures of the *PhPILS* members. **F.** Schematic of amino acids conservation among the moso bamboo *PhPIN.* **G.** Gene structures of the *PhLAX* genes. **H.** Schematic of amino acids conservation among PhLAX proteins. Each gene structure is presented by exons (blue boxes) and introns (black lines between the blue boxes). The alignment profiles of all the proteins were generated using the ClustalW program. The conserved domains are indicated by underlines. The shading indicates identical and conversed amino acid residues.

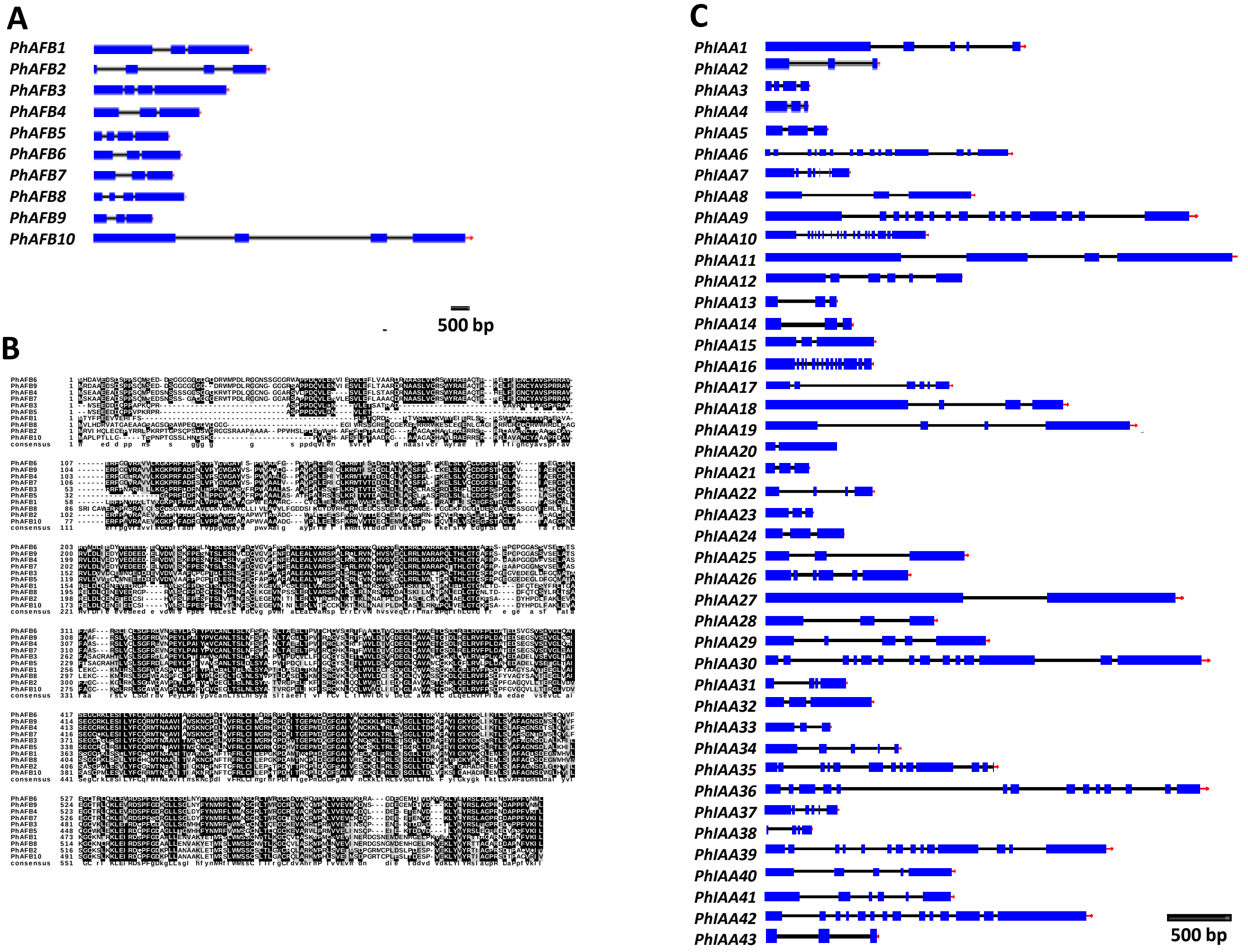
A
PhAFB1
PhAFB2
PhAFB3
PhAFB4
PhAFB5
PhAFB6
PhAFB7
PhAFB8
PhAFB9
PhAFB10
500 bp
B
C
PhIAA1
PhIAA2
PhIAA3
PhIAA4
PhIAA5
PhIAA6
PhIAA7
PhIAA8
PhIAA9
PhIAA10
PhIAA11
PhIAA12
PhIAA13
PhIAA14
PhIAA15
PhIAA16
PhIAA17
PhIAA18
PhIAA19
PhIAA20
PhIAA21
PhIAA22
PhIAA23
PhIAA24
PhIAA25
PhIAA26
PhIAA27
PhIAA28
PhIAA29
PhIAA30
PhIAA31
PhIAA32
PhIAA33
PhIAA34
PhIAA35
PhIAA36
PhIAA37
PhIAA38
PhIAA39
PhIAA40
PhIAA41
PhIAA42
PhIAA43
500 bp

D

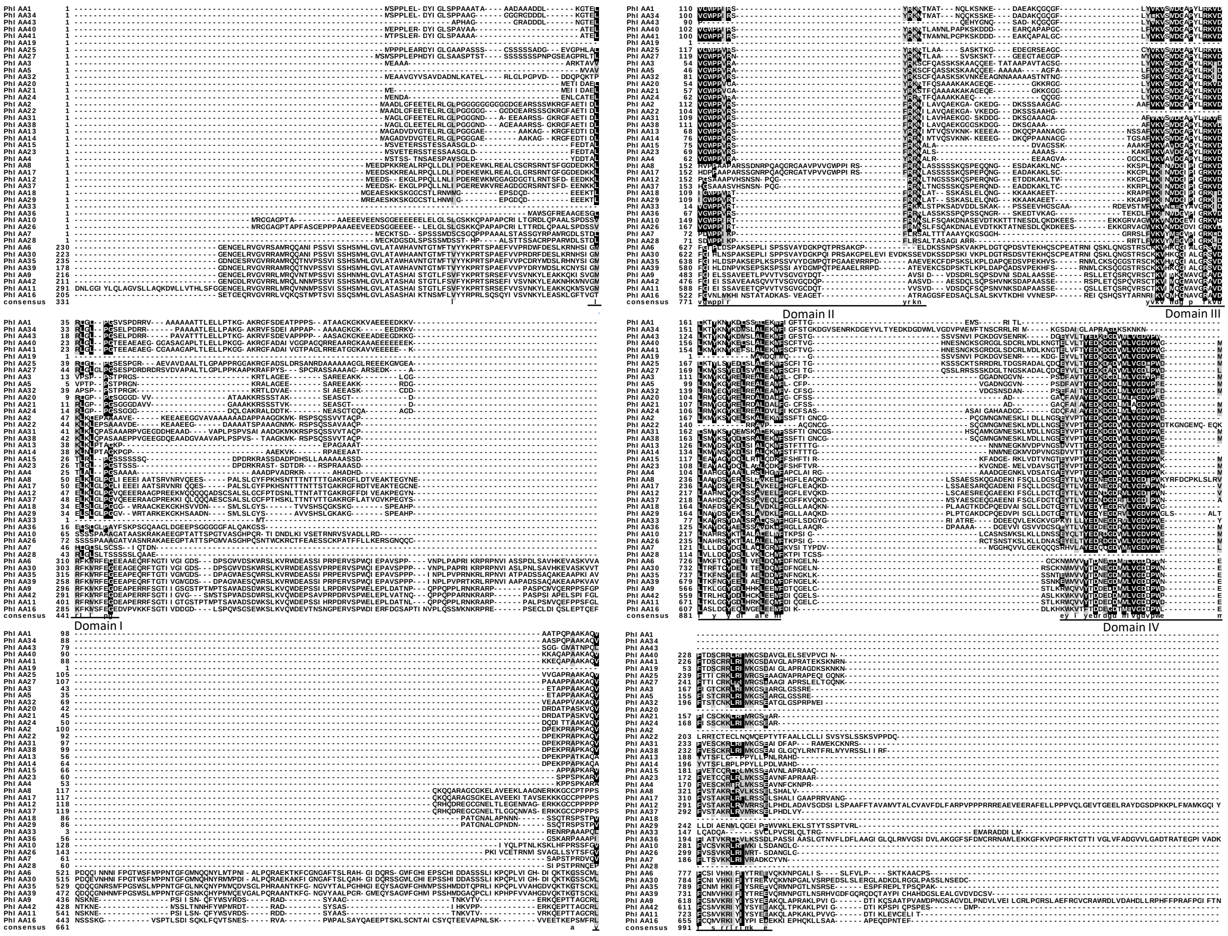

E

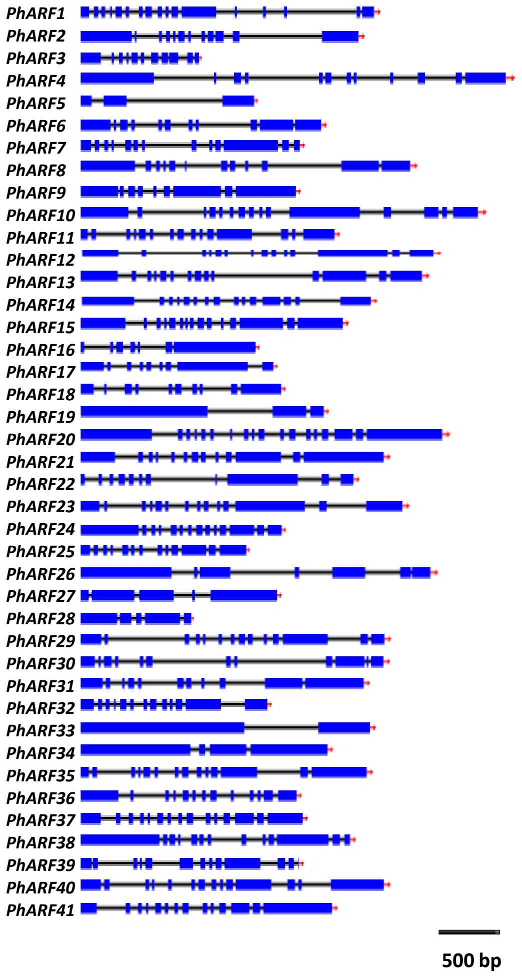
PhARF1
PhARF2
PhARF3
PhARF4
PhARF5
PhARF6
PhARF7
PhARF8
PhARF9
PhARF10
PhARF11
PhARF12
PhARF13
PhARF14
PhARF15
PhARF16
PhARF17
PhARF18
PhARF19
PhARF20
PhARF21
PhARF22
PhARF23
PhARF24
PhARF25
PhARF26
PhARF27
PhARF28
PhARF29
PhARF30
PhARF31
PhARF32
PhARF33
PhARF34
PhARF35
PhARF36
PhARF37
PhARF38
PhARF39
PhARF40
PhARF41
500 bp

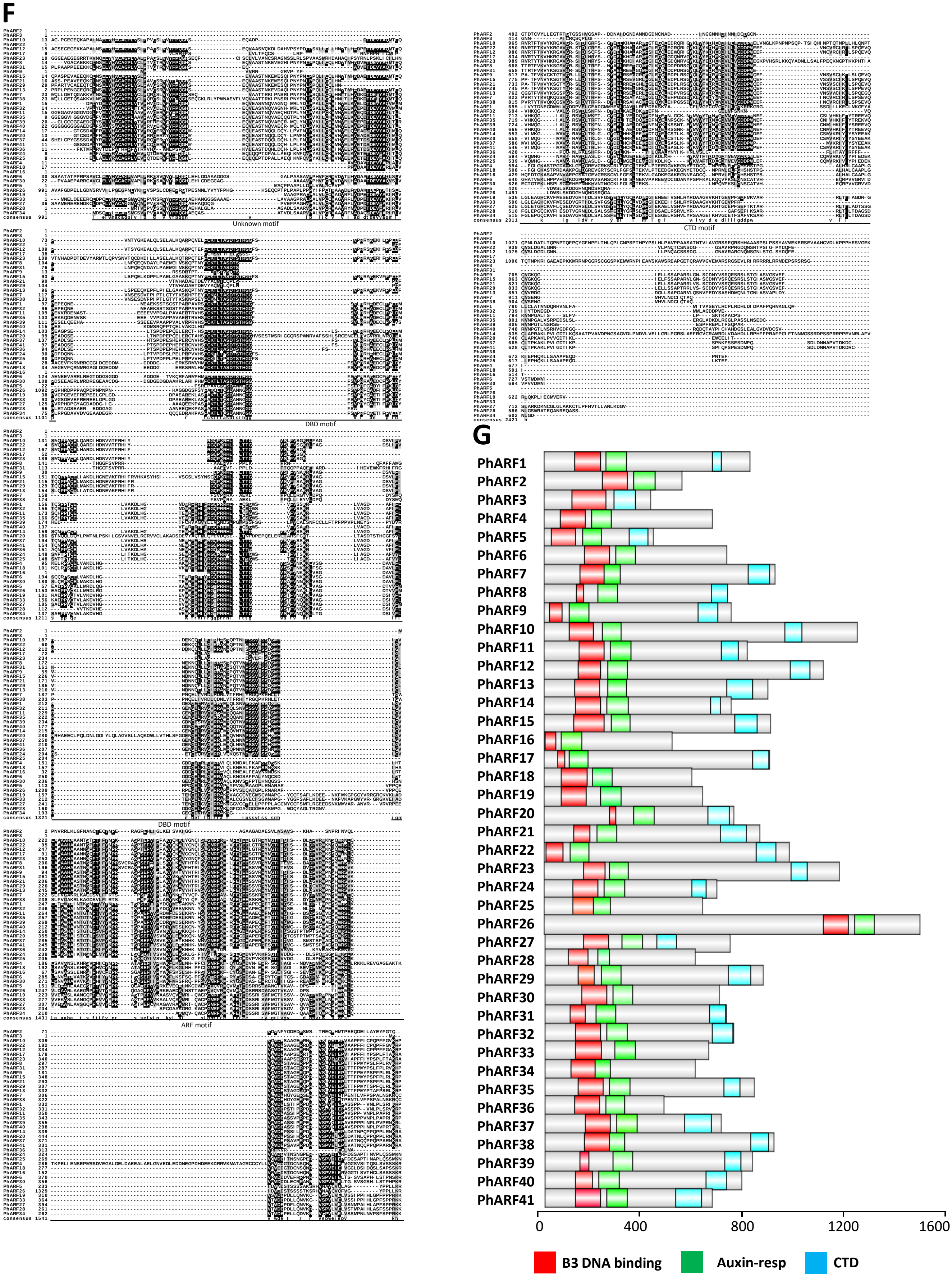
F
Unknown motif
DBD motif
DBD motif
ARF motif
CTD motif
G
PhARF1
PhARF2
PhARF3
PhARF4
PhARF5
PhARF6
PhARF7
PhARF8
PhARF9
PhARF10
PhARF11
PhARF12
PhARF13
PhARF14
PhARF15
PhARF16
PhARF17
PhARF18
PhARF19
PhARF20
PhARF21
PhARF22
PhARF23
PhARF24
PhARF25
PhARF26
PhARF27
PhARF28
PhARF29
PhARF30
PhARF31
PhARF32
PhARF33
PhARF34
PhARF35
PhARF36
PhARF37
PhARF38
PhARF39
PhARF40
PhARF41
0
400
800
1200
1600
B3 DNA binding
Auxin-resp
CTD

Figure S3. Multiple sequence alignment, conserved motif and gene structure analysis of members involved in auxin signaling. **A.** Gene structures of the *PhAFB members*. **B.** Schematic of amino acids conservation among PhAFB proteins. **C.** Gene structures of the *PhIAA members*. **D.** Schematic of amino acids conservation among PhIAA proteins. **E.** Gene structures of the *PhARF members*. **F.** Schematic of amino acids conservation among PhARF proteins. The gene structures of the *PhAFB* (A), *PhIAA* (C), and *PhARF* (E) were presented by exons (blue boxes) and introns (black lines between the blue boxes). Alignment profiles of 10 PhAFBs (B), 43 PhIAAs (D), and 41 PhARFs (F) with ClustalW program were shown. The shading indicates identical and conserved amino acid residues. The typical domains of AUX/IAA and ARF are indicated by underlines. **G.** Domain organization of the PhARF family members. The conserved domains were predicted based on NCBI's conserved domain database. The B3 DNA binding domain, Auxin response domain and the C-terminal domain are shown in red, green and blue, respectively.

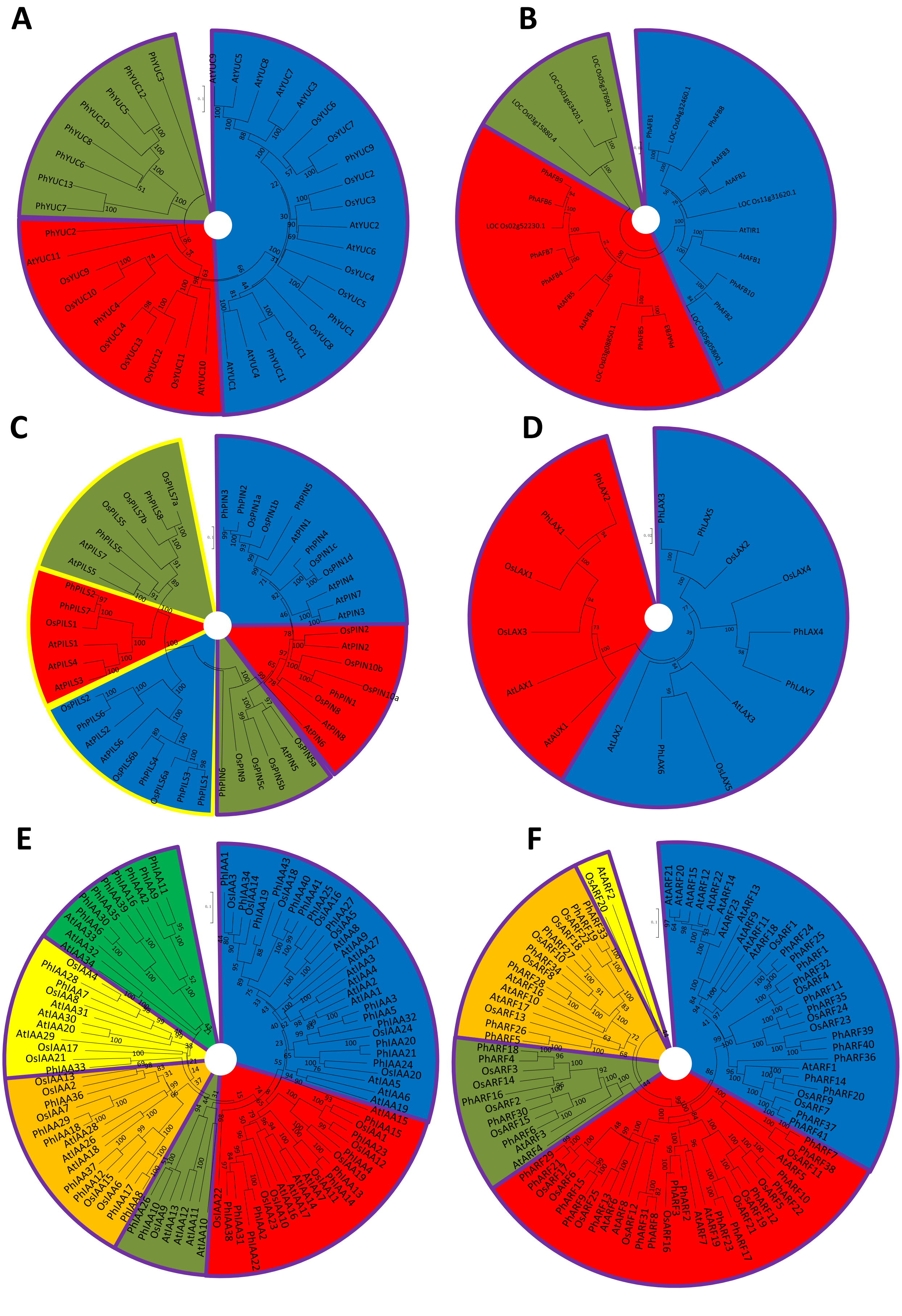

A
AtYUC9
AtYUC5
AtYUC8
AtYUC7
AtYUC3
OsYUC6
OsYUC7
PhYUC9
OsYUC2
OsYUC3
AtYUC2
AtYUC6
OsYUC4
OsYUC5
PhYUC1
OsYUC8
OsYUC1
PhYUC11
AtYUC4
AtYUC1
AtYUC10
OsYUC11
OsYUC12
OsYUC13
OsYUC14
PhYUC4
OsYUC10
OsYUC9
AtYUC11
PhYUC2
PhYUC7
PhYUC13
PhYUC6
PhYUC8
PhYUC10
PhYUC5
PhYUC12
PhYUC3
B
PhAFB1
LOC Os04g32460.1
PhAFB8
AtAFB3
AtAFB2
LOC Os11g31620.1
AtTIR1
AtAFB1
PhAFB10
PhAFB2
LOC Os05g05800.1
PhAFB3
PhAFB5
LOC Os03g08850.1
AtAFB4
AtAFB5
PhAFB4
PhAFB7
LOC Os02g52230.1
PhAFB6
PhAFB9
LOC Os03g15880.4
LOC Os01g63420.1
LOC Os05g37690.1
C
PhPIN3
PhPIN2
OsPIN1a
OsPIN1b
PhPIN5
AtPIN1
PhPIN4
OsPIN1c
OsPIN1d
AtPIN4
AtPIN7
AtPIN3
OsPIN2
AtPIN2
OsPIN10b
OsPIN10a
PhPIN1
OsPIN8
AtPIN8
AtPIN6
OsPIN5a
AtPIN5
OsPIN5b
OsPIN5c
OsPIN9
PhPIN6
PhPILS1
PhPILS3
OsPILS6a
PhPILS4
OsPILS6b
AtPILS6
AtPILS2
PhPILS6
OsPILS2
AtPILS3
AtPILS4
AtPILS1
OsPILS1
PhPILS7
PhPILS2
AtPILS5
AtPILS7
PhPILS5
OsPILS5
OsPILS7b
PhPILS8
OsPILS7a
D
PhLAX3
PhLAX5
OsLAX2
OsLAX4
PhLAX4
PhLAX7
AtLAX3
OsLAX5
PhLAX6
AtLAX2
AtAUX1
AtLAX1
OsLAX3
OsLAX1
PhLAX1
PhLAX2
E
PhIAA1
OsIAA3
PhIAA34
OsIAA14
PhIAA19
PhIAA43
OsIAA18
PhIAA40
PhIAA41
PhIAA25
OsIAA16
PhIAA27
AtIAA5
AtIAA8
AtIAA9
AtIAA27
AtIAA3
AtIAA4
AtIAA2
AtIAA1
PhIAA3
PhIAA5
PhIAA32
OsIAA24
PhIAA20
PhIAA21
PhIAA24
OsIAA20
AtIAA5
AtIAA6
AtIAA19
AtIAA15
PhIAA15
OsIAA1
PhIAA23
OsIAA12
PhIAA4
OsIAA19
PhIAA13
PhIAA14
OsIAA11
AtIAA7
AtIAA14
AtIAA17
AtIAA16
OsIAA10
OsIAA23
PhIAA2
PhIAA22
PhIAA31
PhIAA38
OsIAA22
AtIAA10
AtIAA11
AtIAA12
AtIAA13
OsIAA9
PhIAA10
PhIAA26
PhIAA8
PhIAA17
OsIAA6
OsIAA15
PhIAA12
PhIAA37
AtIAA18
AtIAA26
AtIAA28
PhIAA18
PhIAA29
OsIAA7
PhIAA36
OsIAA2
OsIAA13
PhIAA33
OsIAA21
OsIAA17
AtIAA29
AtIAA20
AtIAA30
AtIAA31
OsIAA8
PhIAA7
PhIAA28
OsIAA4
AtIAA34
AtIAA32
AtIAA33
PhIAA6
PhIAA30
PhIAA35
PhIAA39
PhIAA16
PhIAA42
PhIAA9
PhIAA11
F
AtARF21
AtARF20
AtARF15
AtARF12
AtARF22
AtARF14
AtARF23
AtARF13
AtARF9
AtARF11
AtARF18
OsARF1
PhARF24
PhARF25
PhARF1
PhARF32
OsARF4
PhARF11
PhARF35
OsARF24
OsARF23
PhARF39
PhARF40
PhARF36
AtARF1
PhARF14
PhARF20
OsARF9
OsARF7
PhARF37
PhARF41
PhARF7
PhARF38
OsARF11
AtARF5
PhARF10
PhARF22
OsARF5
OsARF12
OsARF19
OsARF21
PhARF23
PhARF17
AtARF19
AtARF7
PhARF2
OsARF16
PhARF3
PhARF8
PhARF31
OsARF12
AtARF8
AtARF6
PhARF13
OsARF25
PhARF9
PhARF15
OsARF6
OsARF17
PhARF21
PhARF29
AtARF4
AtARF3
PhARF6
OsARF15
PhARF30
OsARF2
PhARF16
OsARF14
OsARF3
PhARF4
PhARF18
PhARF5
PhARF26
OsARF13
AtARF17
AtARF10
AtARF16
PhARF28
OsARF8
PhARF34
OsARF10
PhARF27
OsARF18
OsARF22
PhARF19
PhARF33
OsARF20
AtARF2

Figure S4. Phylogenetic analysis of families related to auxin action in moso bamboo, rice, and Arabidopsis. The protein sequences of YUC family members for auxin biosynthesis **(A)**, AFB proteins for auxin perception **(B)**, PILS and PIN proteins for auxin efflux transport **(C)**, LAX family members for auxin influx transport **(D)**, and AUX/IAA and ARF family members for auxin signaling (**E** and **F**) from moso bamboo, rice and Arabidopsis were derived from BambooGDB, the rice genome annotation database, and the Arabidopsis information resource respectively. The phylogenetic trees were then obtained after alignment of the full-length protein sequences using ClustalW and clustering with the neighbour-joining method. The percentages of replicate trees in which the associated taxa clustered together in the bootstrap test (1000 replicates) are shown next to the branches. The auxin-related genes were classified into different subclasses, and colors are coded according to their classes. Blue, Class I; Red, Class II; Yellow-green, Class III; Orange, Class IV; Yellow, Class V; Green, Class VI.

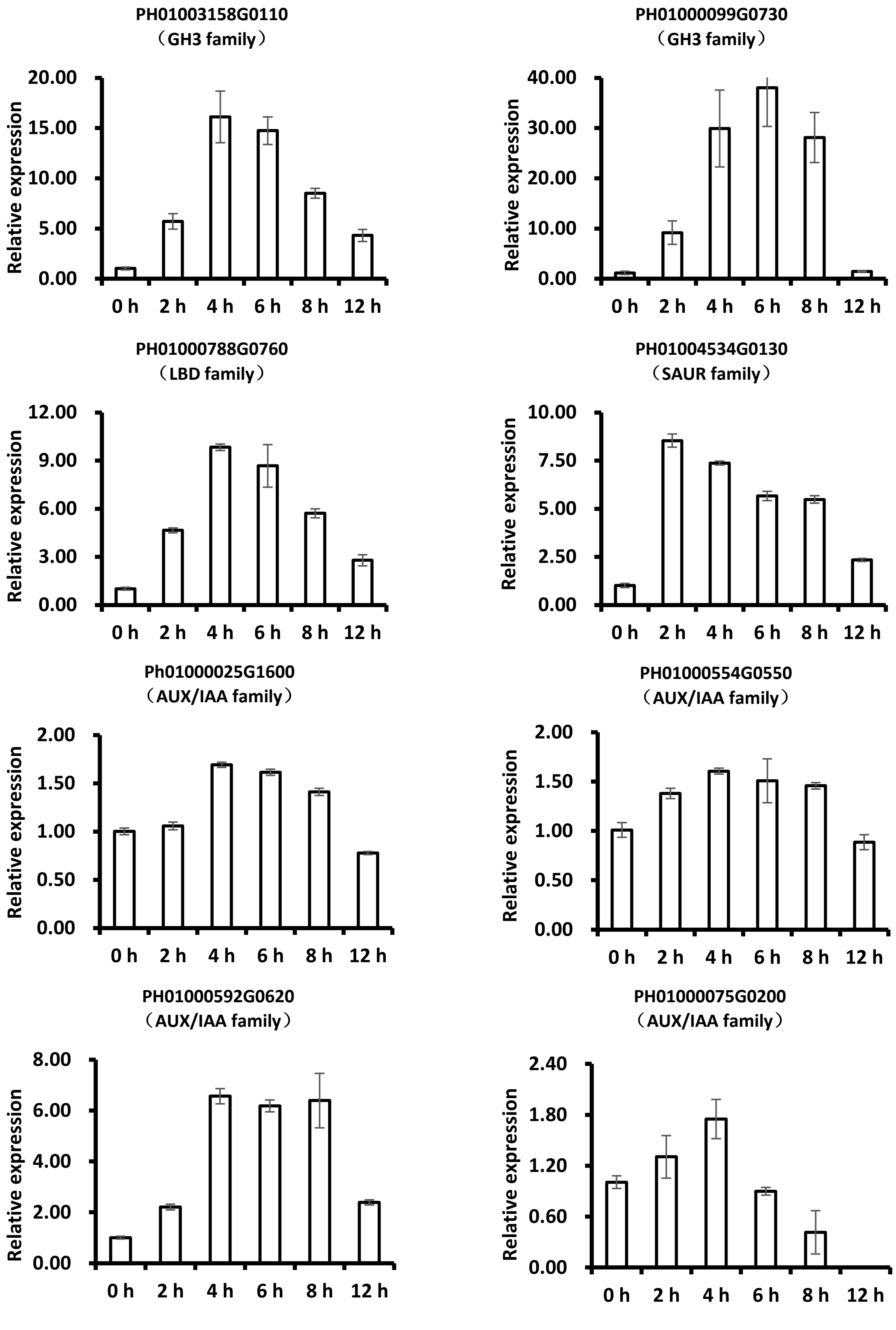
PH01003158G0110
（GH3 family）
Relative expression
20.00
15.00
10.00
5.00
0.00
0 h
2 h
4 h
6 h
8 h
12 h
PH01000099G0730
（GH3 family）
Relative expression
40.00
30.00
20.00
10.00
0.00
0 h
2 h
4 h
6 h
8 h
12 h
PH01000788G0760
（LBD family）
Relative expression
12.00
9.00
6.00
3.00
0.00
0 h
2 h
4 h
6 h
8 h
12 h
PH01004534G0130
（SAUR family）
Relative expression
10.00
7.50
5.00
2.50
0.00
0 h
2 h
4 h
6 h
8 h
12 h
Ph01000025G1600
（AUX/IAA family）
Relative expression
2.00
1.50
1.00
0.50
0.00
0 h
2 h
4 h
6 h
8 h
12 h
PH01000554G0550
（AUX/IAA family）
Relative expression
2.00
1.50
1.00
0.50
0.00
0 h
2 h
4 h
6 h
8 h
12 h
PH01000592G0620
（AUX/IAA family）
Relative expression
8.00
6.00
4.00
2.00
0.00
0 h
2 h
4 h
6 h
8 h
12 h
PH01000075G0200
（AUX/IAA family）
Relative expression
2.40
1.80
1.20
0.60
0.00
0 h
2 h
4 h
6 h
8 h
12 h

Figure S5. qRT-PCR analysis of auxin responsive marker genes in response to exogenous auxin treatment. Samples were collected at the time points indicated in the charts after 5 µM NAA treatment. Total RNA was extracted and the real-time PCR analyses were performed with gene specific primers shown in the charts. Data represents the average of three independent experiments with standard error (SE).

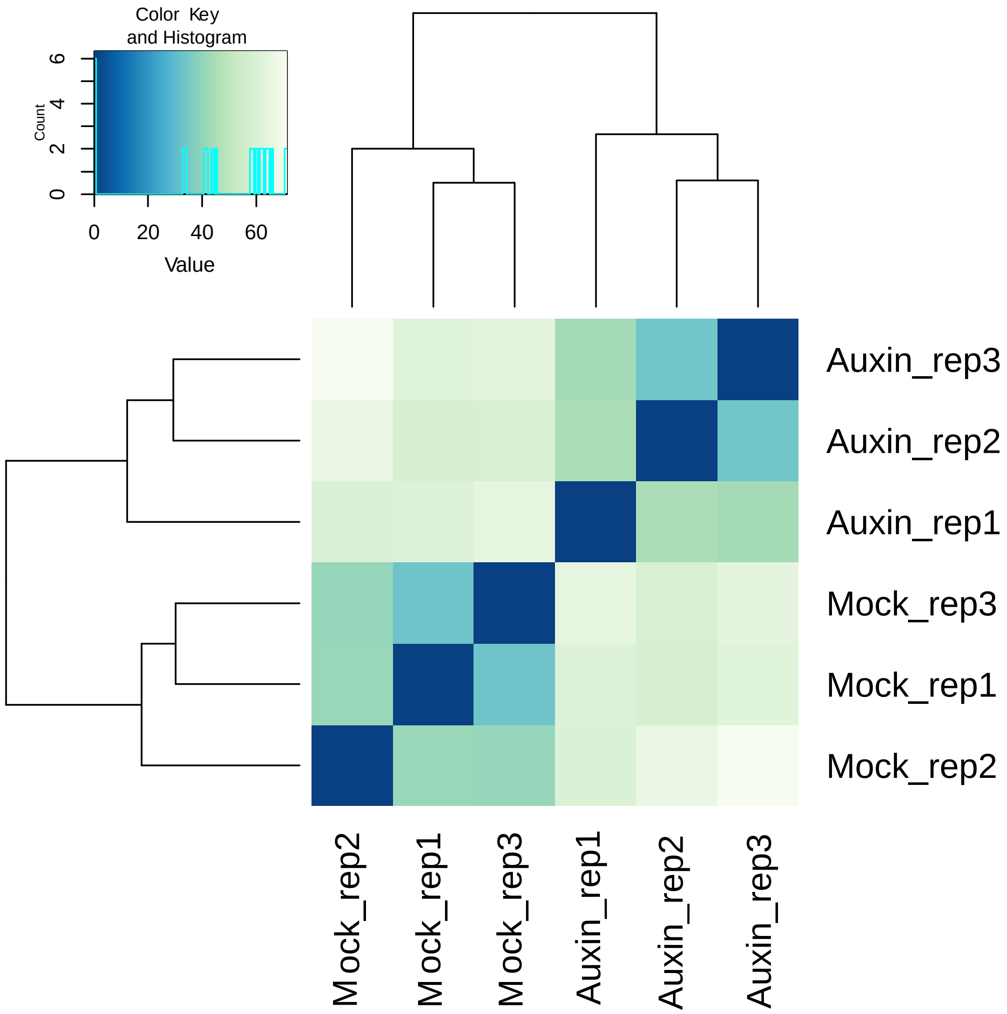

Color Key
and Histogram
Count
0
2
4
6
0
20
40
60
Value
Auxin_rep3
Auxin_rep2
Auxin_rep1
Mock_rep3
Mock_rep1
Mock_rep2
Mock_rep2
Mock_rep1
Mock_rep3
Auxin_rep1
Auxin_rep2
Auxin_rep3

Figure S6. Heat-map of Euclidean distance among 6 bamboo RNA-Seq libraries used in this study. Expression correlation matrix heatmap among six RNA-seq libraries, which were clustered according to Euclidean distance. Strong correlation is shown as darker color.

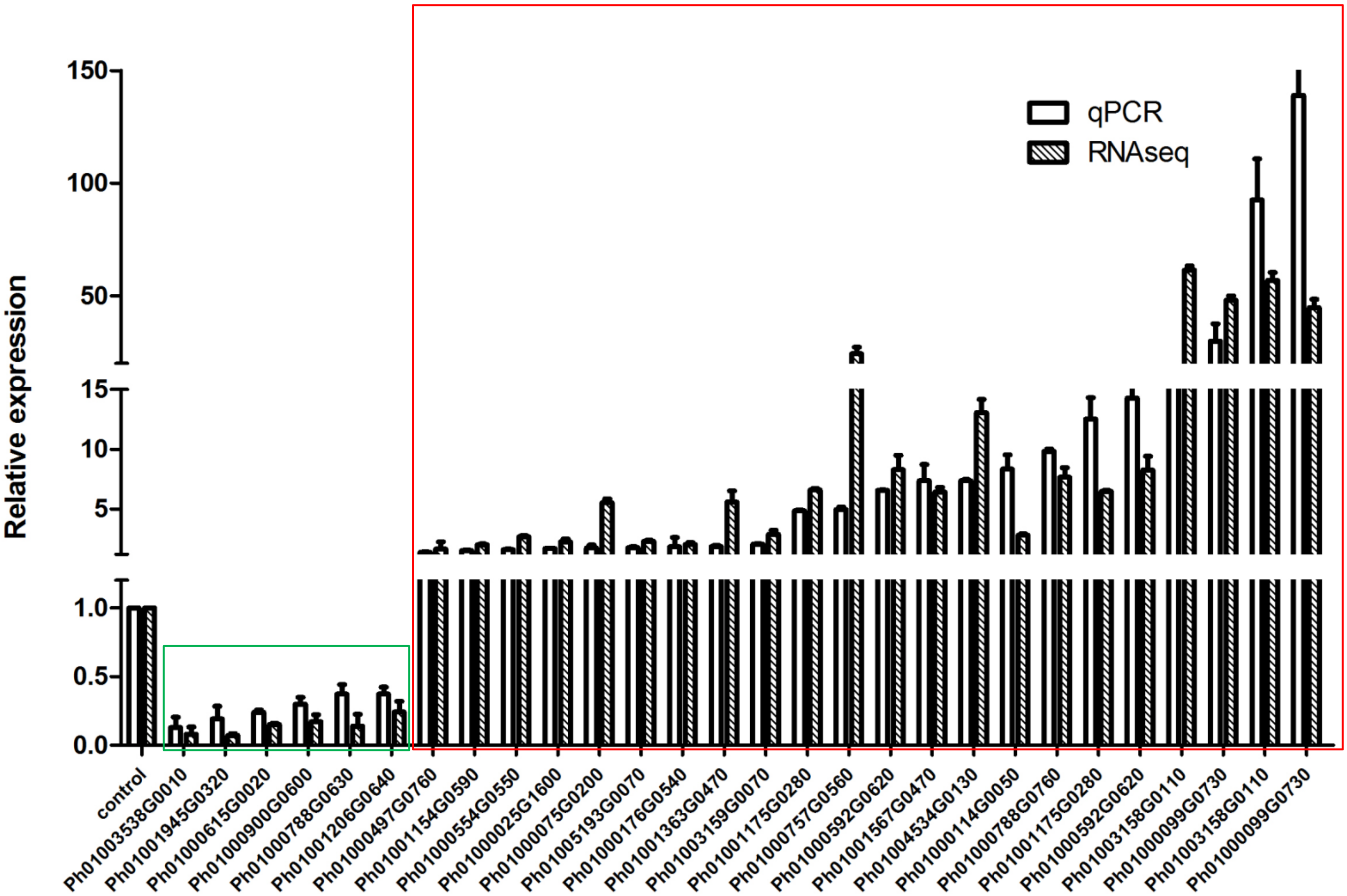

Relative expression
150
100
50
15
10
5
1.0
0.5
0.0
qPCR
RNAseq
control
Ph01003538G0010
Ph01001945G0320
Ph01000615G0020
Ph01000900G0600
Ph01000788G0630
Ph01001206G0640
Ph01000497G0760
Ph01001154G0590
Ph01000554G0550
Ph01000025G1600
Ph01000075G0200
Ph01005193G0070
Ph01000176G0540
Ph01001363G0470
Ph01003159G0070
Ph01001175G0280
Ph01000757G0560
Ph01000592G0620
Ph01001567G0470
Ph01004534G0130
Ph01000114G0050
Ph01000788G0760
Ph01001175G0280
Ph01000592G0620
Ph01003158G0110
Ph01000099G0730
Ph01003158G0110
Ph01000099G0730

Figure S7. Validation of RNA-Seq results by qPCR. 28 differentially expressed genes related to auxin action were selected for qPCR analysis; these results are from 3 biological repeats. Green rectangle, down-regulated genes; Red rectangle, up-regulated genes.

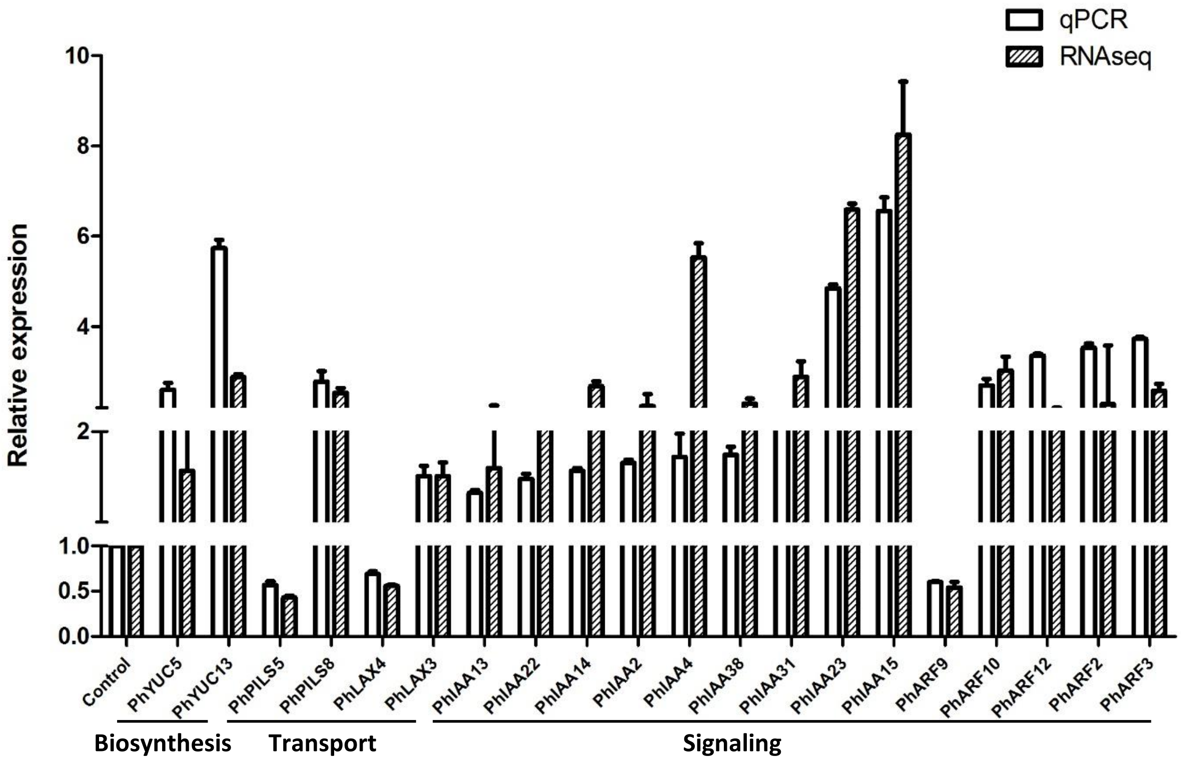
qPCR
RNAseq
Relative expression
10
8
6
4
2
1.0
0.5
0.0
Control
PhYUC5
PhYUC13
PhPILS5
PhPILS8
PhLAX4
PhLAX3
PhIAA13
PhIAA22
PhIAA14
PhIAA2
PhIAA4
PhIAA38
PhIAA31
PhIAA23
PhIAA15
PhARF9
PhARF10
PhARF12
PhARF2
PhARF3
Biosynthesis
Transport
Signaling

Figure S8. qRT-PCR confirmation of the expressions of auxin-related genes. Auxin related genes with changed expression (including members from auxin biosynthesis, transport and signaling pathways) were randomly selected from different gene families for qPCR analysis. And the consistence between RNA-Seq and qRT-PCR were shown.

A

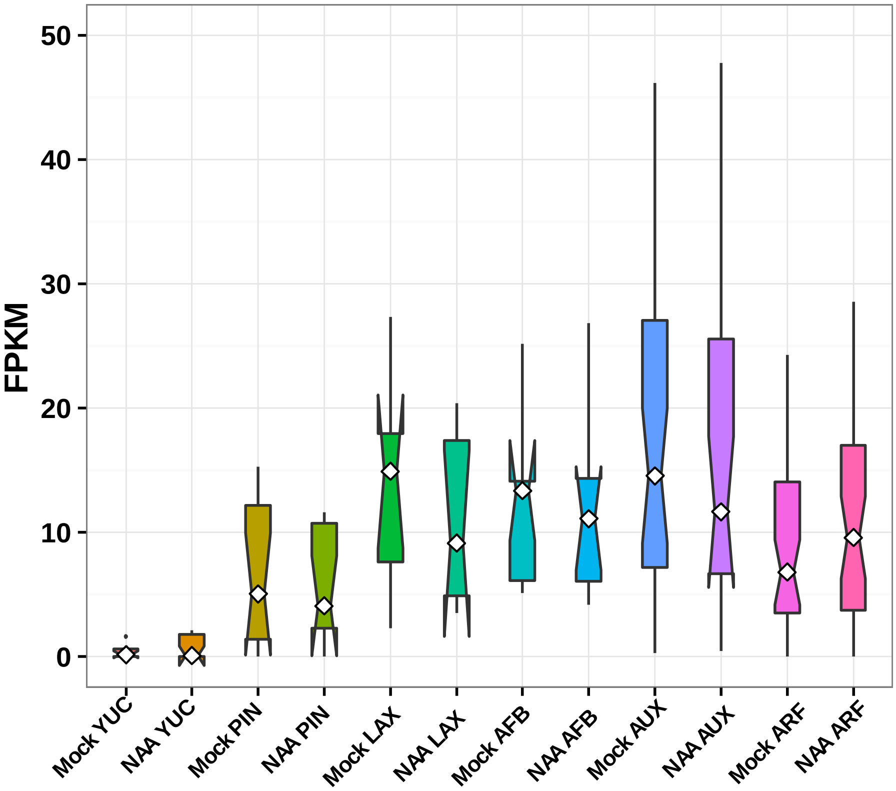

FPKM
50
40
30
20
10
0
Mock YUC
NAA YUC
Mock PIN
NAA PIN
Mock LAX
NAA LAX
Mock AFB
NAA AFB
Mock AUX
NAA AUX
Mock ARF
NAA ARF

B

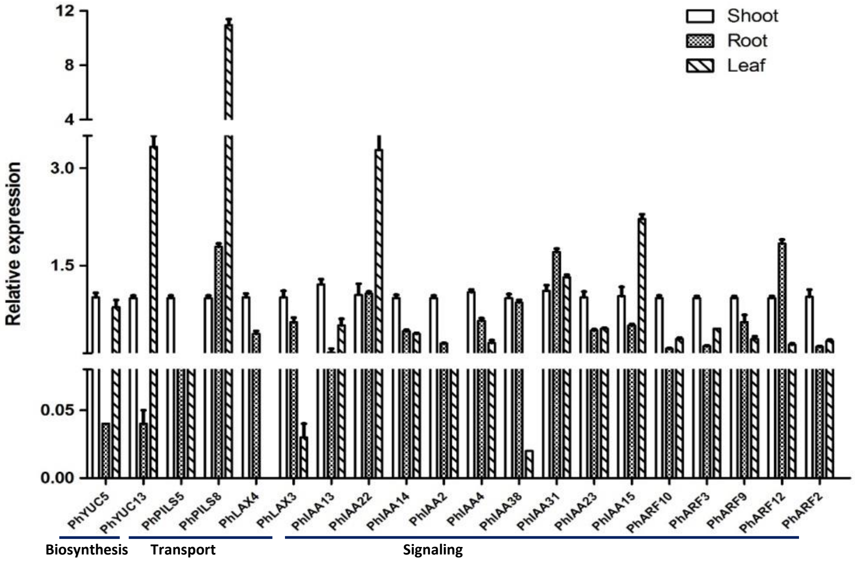

Shoot
Root
Leaf
Relative expression
12
8
4
3.0
1.5
0.05
0.00
PhYUC5
PhYUC13
PhPILS5
PhPILS8
PhLAX4
PhLAX3
PhIAA13
PhIAA22
PhIAA14
PhIAA2
PhIAA4
PhIAA38
PhIAA31
PhIAA23
PhIAA15
PhARF10
PhARF3
PhARF9
PhARF12
PhARF2
Biosynthesis
Transport
Signaling

Figure S9. Tissue expression patterns of the auxin related genes. **A**. Represented boxplot of FPKM from the control and auxin treatment genes of six different families, respectively. **B.** The expression patterns of selected auxin related genes from different families were tested by qRT-PCR. Samples (leaf, root and shoot) were collected from 1-month old bamboo seedlings grown in the greenhouse. Total RNA was extracted and the real-time PCR analyses were performed with gene-specific primers shown in the charts. Data represents the average of three independent experiments $\pm$ SE.

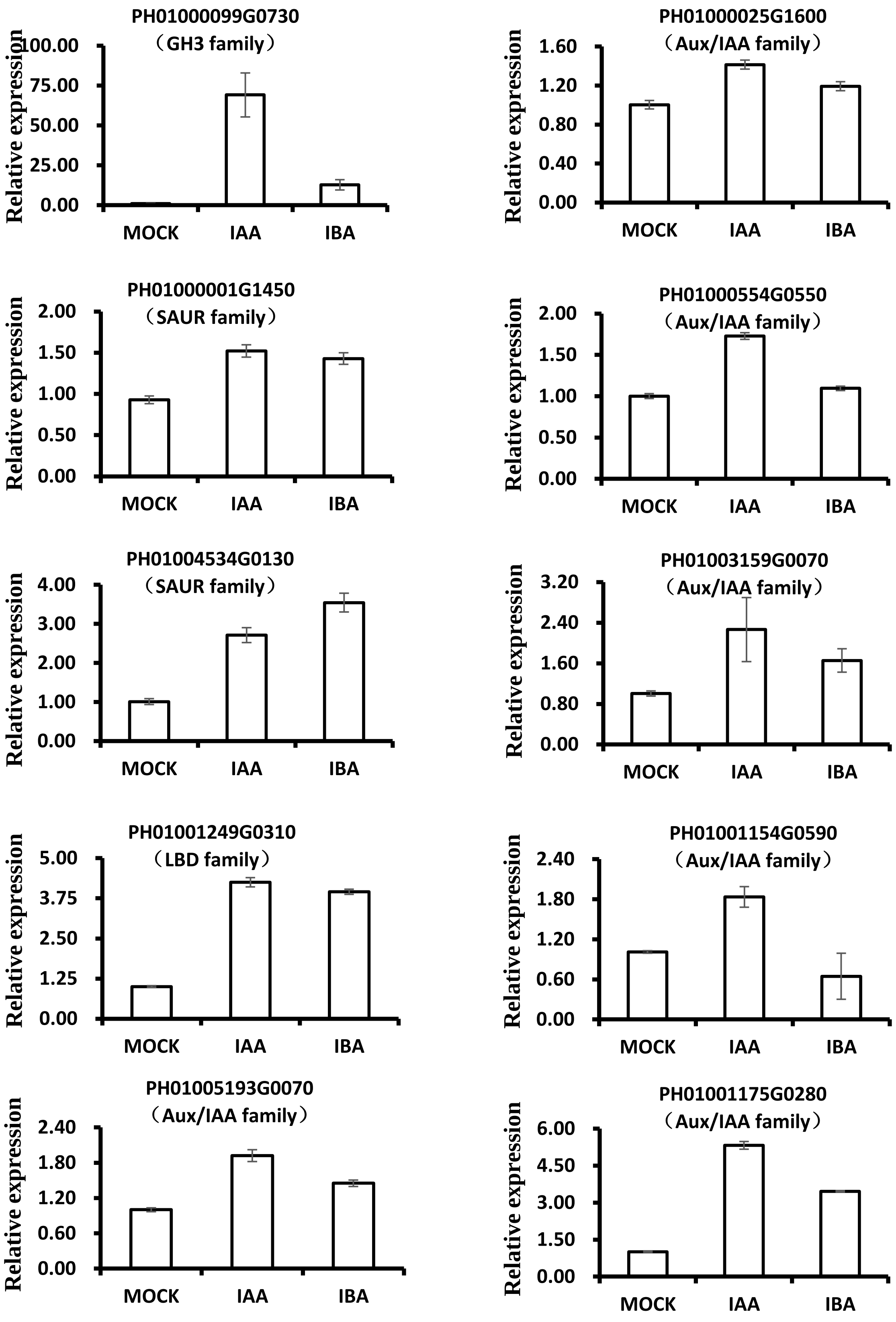
PH01000099G0730
（GH3 family）
Relative expression
100.00
75.00
50.00
25.00
0.00
MOCK
IAA
IBA
PH01000025G1600
（Aux/IAA family）
Relative expression
1.60
1.20
0.80
0.40
0.00
MOCK
IAA
IBA
PH01000001G1450
（SAUR family）
Relative expression
2.00
1.50
1.00
0.50
0.00
MOCK
IAA
IBA
PH01000554G0550
（Aux/IAA family）
Relative expression
2.00
1.50
1.00
0.50
0.00
MOCK
IAA
IBA
PH01004534G0130
（SAUR family）
Relative expression
4.00
3.00
2.00
1.00
0.00
MOCK
IAA
IBA
PH01003159G0070
（Aux/IAA family）
Relative expression
3.20
2.40
1.60
0.80
0.00
MOCK
IAA
IBA
PH01001249G0310
（LBD family）
Relative expression
5.00
3.75
2.50
1.25
0.00
MOCK
IAA
IBA
PH01001154G0590
（Aux/IAA family）
Relative expression
2.40
1.80
1.20
0.60
0.00
MOCK
IAA
IBA
PH01005193G0070
（Aux/IAA family）
Relative expression
2.40
1.80
1.20
0.60
0.00
MOCK
IAA
IBA
PH01001175G0280
（Aux/IAA family）
Relative expression
6.00
4.50
3.00
1.50
0.00
MOCK
IAA
IBA

Figure S10. qRT-PCR analysis of auxin responsive marker genes in response to exogenous IAA and IBA treatment. Samples were collected at 4 h after 5 μM NAA treatment. Total RNA was extracted and the real-time PCR analyses were performed with gene-specific primers shown in the charts. Data represents the average of three independent experiments ± SE.
